# Ultralong room-temperature phosphorescence of a solid-state supramolecule between phenylmethylpyridinium and cucurbit[6]uril†
†Electronic supplementary information (ESI) available. See DOI: 10.1039/c9sc02633a


**DOI:** 10.1039/c9sc02633a

**Published:** 2019-07-01

**Authors:** Zhi-Yuan Zhang, Yu Liu

**Affiliations:** a Department of Chemistry , State Key Laboratory of Elemento-Organic Chemistry , Nankai University , Tianjin 300071 , P. R. China . Email: yuliu@nankai.edu.cn

## Abstract

Long-lived organic room-temperature phosphorescence (RTP) has received great attention because of its various potential applications. Herein, we report a persistent RTP of a solid-state supramolecule between a cucurbit[6]uril (CB[6]) host and a heavy-atom-free phenylmethylpyridinium guest. Significantly, the long-lived phosphorescence completely depends on the host–guest complexation, revealing that the non-phosphorescent guest exhibits a 2.62 s ultralong lifetime after being complexed by CB[6] under ambient conditions. The ultralong RTP is because of tight encapsulation of CB[6], which boosts intersystem crossing, suppresses nonradiative relaxation and possibly shields quenchers. Moreover, several phosphorescent complexes possessing different lifetimes are prepared and successfully applied in triple lifetime-encoding for data encryption and anti-counterfeiting. This strategy provides a new insight for realizing purely organic RTP with ultralong lifetime and expands its application in the field of information protection.

## Abstract 2

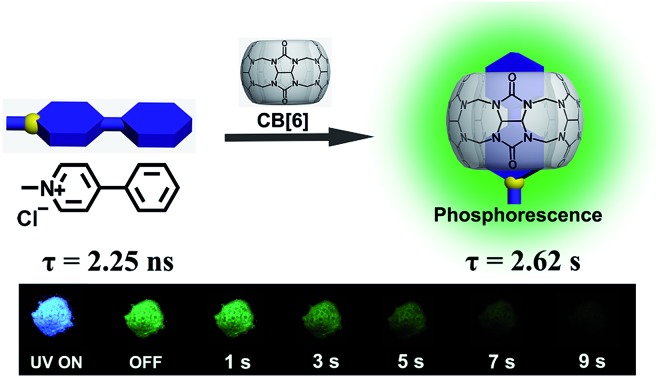
We report an ultralong room-temperature phosphorescence (2.62 s) based on cucurbit[6]uril host and phenyl-methyl-pyridinium guest for data encryption. Encapsulation of CB[6] boosts intersystem crossing and suppresses nonradiative relaxation of guest.

## Introduction

Organic room-temperature phosphorescence (RTP) with persistent luminescence has received enormous attention because of its various applications in biological imaging,^1^,^2^ light-emitting devices,^3^ information storage and encryption,^4^,^5^ and so on. However, achieving ultralong RTP (generally from seconds to hours) is extremely difficult due to two main issues: (i) the intrinsically weak spin–orbit coupling of purely organic molecules makes it hard to achieve efficient intersystem crossing (ISC); (ii) a long triplet state (T_1_) lifetime means that T_1_ will suffer tremendously from quenching by a very low concentration of impurities even in crystals.^6^ Several methods have been reported to actualize long-lived RTP, such as forming charge-separated states,^7^ crystallization,^8^–^17^ polymerization,^18^–^21^ embedding into suitable matrices,^22^–^24^ and so on.^25^–^27^ Recently, Huang and co-workers achieved lifetimes of 1.35 s and 1.91 s by reasonable crystal design.^28^,^29^ Fukushima and Nakai *et al.* found that the lifetime of arylboronic esters in crystals could reach 1.85 s due to an appropriate molecular packing.^30^ Zhao and co-workers reported ultralong RTP with a lifetime of 0.75 s by embedding phosphors into polymers.^31^ Tian and co-workers developed purely organic amorphous polymers with a lifetime of 0.537 s by utilizing hydrogen-bonding networks between polymeric chains, and prepared cyclodextrin based phosphors.^32^–^34^ Wu and co-workers reported self-assembled nanoparticles with RTP for cell imaging.^35^ Besides, theoretical descriptors were also utilized to assist in the molecular design of efficient and long-lived RTP.^36^

**Scheme 1:**
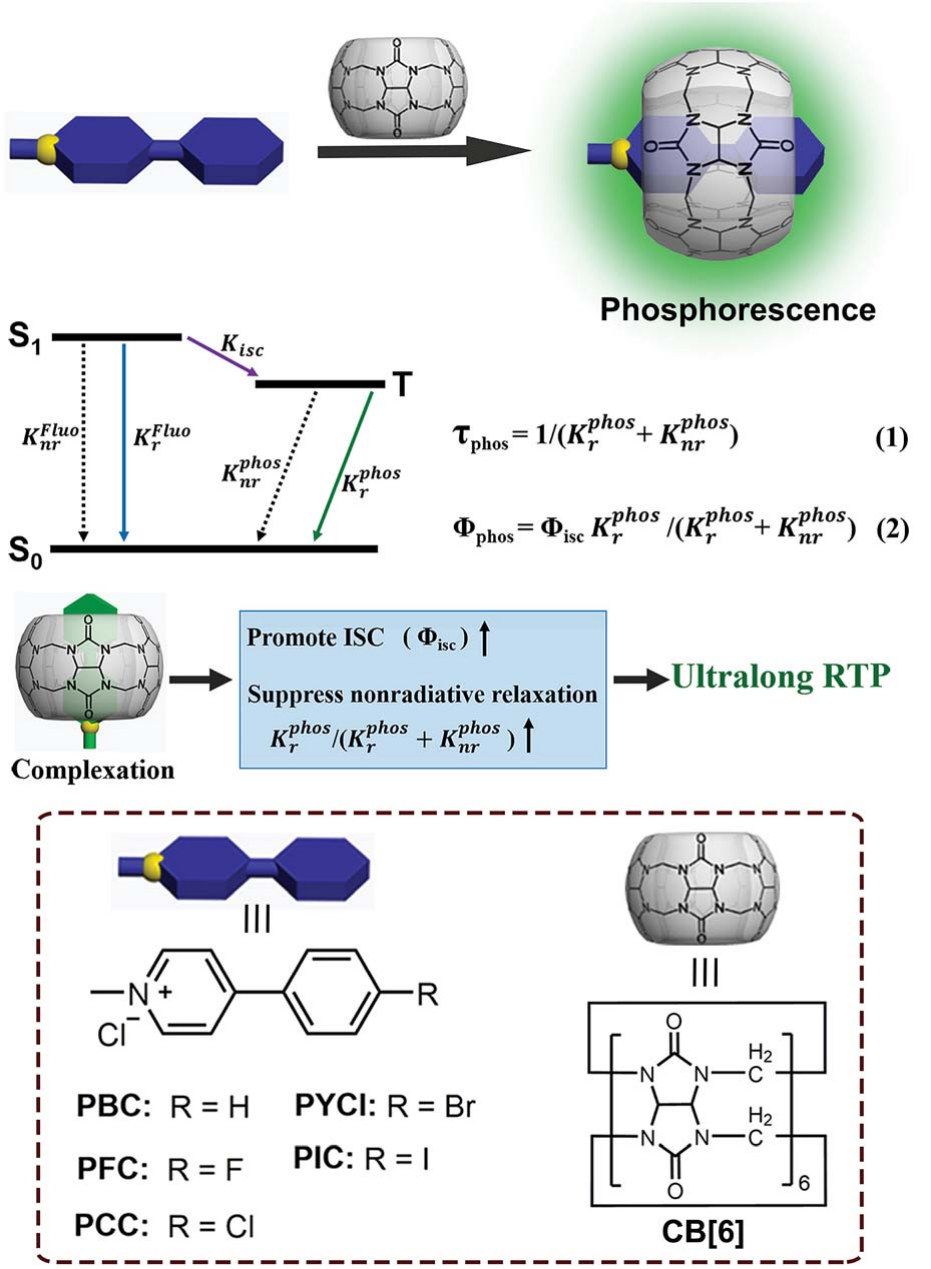
Schematic illustration of the solid-state supramolecular strategy and Jablonski diagram for radiative and non-radiative processes. (*K*Fluor is the radiative rate constant of the lowest excited singlet state S_1_; *K*Fluonr is the nonradiative rate constant of the lowest excited singlet S_1_; *K*_isc_ is the intersystem crossing rate constant from the excited singlet state to the triplet state; *K*Phosr is the radiative rate constant of the lowest excited triplet state T_1_; *K*Phosnr is the nonradiative rate constant of the lowest excited triplet state T_1_; *τ*_Phos_ is the lifetime of the excited triplet state; *Φ*_Phos_ is the quantum yield of phosphorescence; and *Φ*_isc_ is the quantum yield of intersystem crossing from the excited singlet state to the triplet state; inset: the molecular structures of the guests and host).

More recently, we found that the phosphorescence quantum yield of bromophenyl-methyl-pyridinium chloride (PYCl) could reach 81.2% after being complexed by cucurbit[6]uril (CB[6]).^37^ In general, the introduction of heavy atoms facilitates ISC and thus contributes to high phosphorescence quantum yield, but the heavy atoms will result in a shorter lifetime (normally below a millisecond) because of the acceleration of radiative and non-radiative decay rates of the triplet state.^6^ The high efficiency and short lifetime (*τ* = 5.40 ms) of the complex (PYCl/CB[6]) in our recent work is a typical case.^37^ Therefore, removing a heavy atom (Br) from PYCl will slow down radiative and non-radiative decay of the triplet state and probably achieve long-lived RTP. It is reasonable to realize this proposal by a solid-state supramolecular strategy because the aforementioned issues in achieving ultralong RTP will no longer exist: firstly, the encapsulation of CB[6] promotes ISC of the guest and thus boosts the production of triplet states, which will (partially) offset the absence of the heavy atom; secondly, the extreme restriction of the guest by CB[6] will greatly suppress molecular motions (vibrations, rotations, and inter-collisions) and prolong the lifetime of phosphorescence; thirdly, CB[6] will act as a fine shell to prevent quenchers (oxygen, impurities and so on) from attacking triplet states. To verify our hypothesis, phenylmethylpyridinium chloride (PBC) was designed and synthesized. Furthermore, a series of derivatives were prepared to explore the generalizability of the solid-state supramolecular strategy and uncover the relationship between the structure of guests and the phosphorescence properties of complexes (Scheme 1 and Fig. S1–S14†).

## Experimental

### Materials and methods

All chemicals were commercially available unless noted otherwise. NMR spectroscopy was performed on a Bruker AV400 spectrometer. XRD patterns obtained at Rigaku SmartLab 3 kW. High-resolution mass spectrometry (HR-MS) was performed on a Q-TOF LC-MS in ESI/MALDI mode. Photoluminescence spectra and lifetimes were measured on an FLS900 and FLS1000. Phosphorescence quantum efficiencies were measured on a HAMAMATSU C9920-02.

### Synthesis of PX

Compound PBC was synthesized according to the literature.^38^ 4-Phenylpyridine (1.09 g, 5.85 mmol) and methyl iodide (2 mL, 30 mmol) were dissolved in anhydrous ethanol (50 mL) and then heated under reflux for 24 h. After being cooled to room temperature, the reaction mixture was concentrated and the resulting pale gold precipitate was washed with dichloromethane and then dried under vacuum to yield a pale gold solid (1.32 g, 76%). After dispersing the solid in 30 mL acetonitrile, saturated aqueous NH_4_PF_6_ was added until all of the solid dissolved. After evaporating the acetonitrile and filtering, a white solid was obtained. After dissolving the solid in CH_3_CN, tetrabutylammonium chloride was added until no solid appeared any more. PBC (white solid) was obtained after filtering and washing with CH_3_CN (10 mL × 3). ^1^H NMR (400 MHz, DMSO) *δ* 9.12 (d, *J* = 6.2 Hz, 2H), 8.53 (d, *J* = 6.3 Hz, 2H), 8.09 (d, *J* = 5.6 Hz, 2H), 7.64 (d, *J* = 5.8 Hz, 3H), 4.38 (s, 3H); ^13^C NMR (101 MHz, DMSO) *δ* 154.16, 145.68, 133.54, 131.99, 129.64, 128.05, 124.06, 46.96.

Compound PFC was synthesized according to the literature.^38^ Cesium carbonate (0.98 g, 3.0 mmol) and tetrakis(triphenylphosphine)palladium (0.23 g, 0.20 mmol) were added to a stirred solution of 4-bromopyridine hydrochloride (0.49 g, 2.5 mmol) and 4-fluorobenzeneboronic acid (0.42 g, 3.0 mmol) in dioxane/methanol (125 mL). The mixture was heated under reflux for 24 h and then concentrated. The slurry was triturated with ethyl acetate (40 mL) and the organic phase was washed with saturated aqueous sodium bicarbonate (30 mL × 3), water (30 mL × 2), and brine (100 mL), and then dried over sodium sulfate. After removal of the solvent by evaporation, the resulting residue was subjected to column chromatography (petroleum ether/ethyl acetate 8 : 1) to give a white solid. Then the solid reacted with methyl iodide and was purified in a similar manner to PBC. After ion exchange, PFC was obtained as a white solid. ^1^H NMR (400 MHz, D_2_O) *δ* 8.69 (d, *J* = 6.5 Hz, 2H), 8.16 (d, *J* = 6.5 Hz, 2H), 7.88 (dd, *J* = 8.6, 5.3 Hz, 2H), 7.28 (t, *J* = 8.7 Hz, 2H), 4.32 (s, 3H); ^13^C NMR (101 MHz, D_2_O) *δ* 166.16, 163.66, 154.96, 144.81, 130.35, 124.44, 116.75, 47.17; HRMS (*m*/*z*): [M–Cl]^+^ calcd for C_12_H_11_NF^+^, 188.0875; found, 188.0874.

Compound PCC was prepared as a white powder according to a procedure similar to that described for compound PBC: ^1^H NMR (400 MHz, DMSO) *δ* 9.10 (d, *J* = 6.8 Hz, 2H), 8.54 (d, *J* = 6.9 Hz, 2H), 8.13 (d, *J* = 8.6 Hz, 2H), 7.72 (d, *J* = 8.6 Hz, 2H), 4.36 (s, 3H); ^13^C NMR (101 MHz, DMSO) *δ* 152.92, 145.77, 137.12, 132.42, 129.96, 129.69, 124.13, 47.08.

Compound PIC was prepared as a pale yellow powder according to a procedure similar to that described for compound PFC: ^1^H NMR (400 MHz, D_2_O) *δ* 8.70 (d, *J* = 6.5 Hz, 2H), 8.15 (d, *J* = 6.5 Hz, 2H), 7.89 (d, *J* = 8.4 Hz, 2H), 7.55 (d, *J* = 8.4 Hz, 2H), 4.33 (s, 3H); ^13^C NMR (101 MHz, D_2_O) *δ* 154.96, 144.92, 138.72, 133.03, 129.22, 124.38, 98.87, 47.33; HRMS (*m*/*z*): [M–Cl]^+^ calcd for C_12_H_11_NI^+^, 295.9936; found, 295.9934.

Compound PEC: ^1^H NMR (400 MHz, DMSO) *δ* 8.93 (d, *J* = 6.8 Hz, 2H), 8.26 (d, *J* = 6.8 Hz, 2H), 8.05 (d, *J* = 16.4 Hz, 1H), 7.76 (d, *J* = 6.8 Hz, 2H), 7.59–7.42 (m, 4H), 4.28 (s, 3H); ^13^C NMR (101 MHz, DMSO) *δ* 152.43, 145.20, 140.58, 135.18, 130.39, 129.14, 128.12, 123.59, 123.36, 46.89.

Compound PC: ^1^H NMR (400 MHz, DMSO) *δ* 9.13 (d, *J* = 5.8 Hz, 2H), 8.59 (t, *J* = 7.8 Hz, 1H), 8.15 (t, *J* = 7.1 Hz, 2H), 4.41 (s, 3H); ^13^C NMR (101 MHz, DMSO) *δ* 145.66, 145.05, 127.67, 47.81.

## Results and discussion

**Fig. 1:**
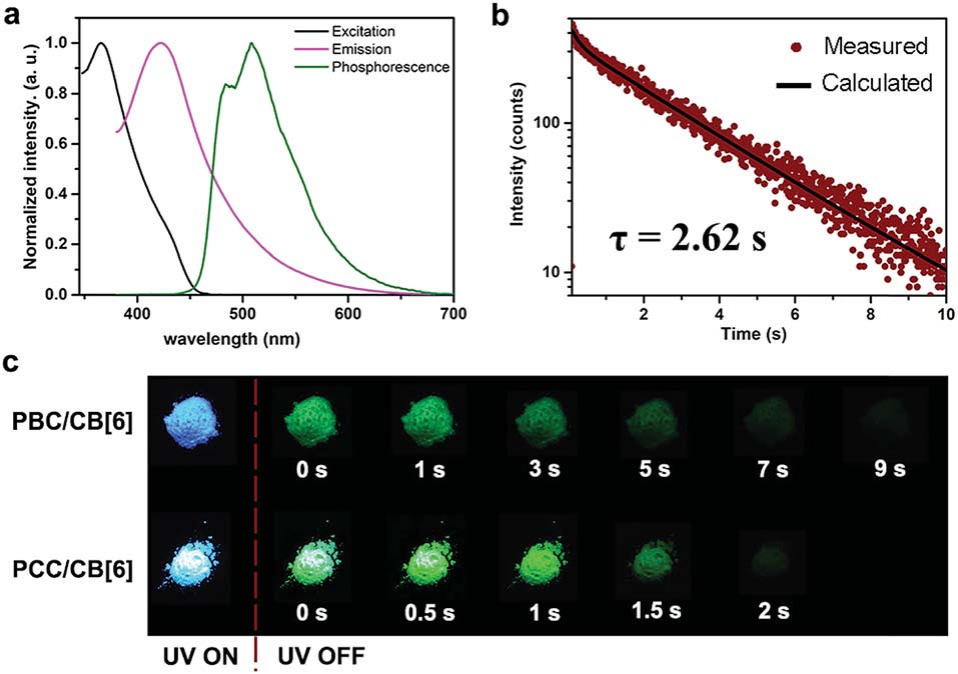
Photophysical properties of PX/CB[6]. (a) Excitation (black), photoluminescence (magenta) and phosphorescence spectra (olive) of PBC/CB[6] in the solid state; (b) time resolved PL decay of PBC/CB[6] at 510 nm in the solid state at room temperature; (c) luminescence photographs of PBC/CB[6] and PCC/CB[6] powder under 365 nm UV irradiation and at different time intervals after removal of the ultraviolet lamp.

Using this strategy, we successfully realized ultralong RTP. The chromophore PBC itself emitted only blue fluorescence (*λ*_max_ = 441 nm) with a lifetime (*τ*) of 2.25 ns (Fig. S15†). Notably, after forming the PBC/CB[6] complex by a grinding method,^37^ the solid emitted a delayed luminescence (*λ*_max_ = 510 nm) with an ultralong phosphorescence of 2.62 s, along with fluorescence at *λ*_max_ = 427 nm (*τ* = 8.40 ns) (Fig. 1a, b and S16†). And the afterglow could last more than 9 s after turning off the UV lamp (Fig. 1c and Video 1†). This excellent long luminescence verified our hypothesis that the removal of the heavy atom could prolong the lifetime of the complex and demonstrated that the complexation of CB[6] could “turn on” the phosphorescence of PBC. Furthermore, the decent phosphorescence quantum yield (9.7%) indicated that the encapsulation of CB[6] promoted ISC of the guest even without the assistance of the heavy atom (Table 1 and Fig. S17b†). Indeed, the intersystem crossing rate (*K*_isc_) was as high as 1.2 × 10^7^ s^–1^. Considering that there was no heavy atom to promote ISC, the result, which was only one magnitude smaller than that of PYCl/CB[6] (2.2 × 10^8^ s^–1^), was satisfactory. Significantly, the radiative decay rate of phosphorescence (*K*Phosr) was very low (3.7 × 10^–2^ s^–1^), about 4000-fold lower than that of PYCl/CB[6] (150 s^–1^), revealing that the absence of the heavy atom would slow down the decay of T_1_ and favor long lifetimes. Normally, a long T_1_ lifetime means that T_1_ will suffer tremendously from quenching by a very low concentration of impurities because of triplet energy migration, so that extremely pure samples and complete exclusion of oxygen are needed in order to observe ultralong RTP.^6^ However, these rigorous conditions were no longer necessary in our case; the PBC/CB[6] powder showed quite robust persistent RTP even upon being exposed to ambient conditions. Two reasons were proposed for this property: (i) CB[6] played the role of a tight shell to prevent quenchers (oxygen, impurities and so on) from attacking the chromophoric core (PBC); (ii) every PBC molecule possessed an individual micro-circumstance provided by CB[6]. The large outer diameter (14.4 Å)^39^ and high rigidity of CB[6] made each complex an independent phosphor unit. Possible defects (empty CB[6] or free PBC) would not produce serious triplet energy migration and therefore had little effect on the whole phosphorescence. They jointly stabilized the triplet state and guaranteed the robust persistent RTP.

**Table 1:** Photophysical data of PX/CB[6]

Compound	E_X_ (nm)	*λ*_Fluo_ (nm)	*λ*_Phos_ (nm)	*τ*_Fluo_ (ns)	*τ*_Phos_ (ms)	*Φ*_Fluo_ (%)	*Φ*_Phos_ (%)	*K* Fluo r ^*a*^ (s^–1^)	*K* Fluo nr ^*b*^ (s^–1^)	*K*_isc_ ^*c*^ (s^–1^)	*K* Phos r ^*d*^ (s^–1^)	*K* Phos nr ^*e*^ (s^–1^)
PBC/CB[6]	366	427	510	8.40	2620	17.3	9.7	2.1 × 10^7^	8.7 × 10^7^	1.2 × 10^7^	3.7 × 10^–2^	0.34
PFC/CB[6]	330	434	520	5.61	0.0095	27.7	1.9	4.9 × 10^7^	1.2 × 10^8^	1.4 × 10^7^	2.0 × 10^3^	1.0 × 10^5^
PCC/CB[6]	370	420	500	2.52	275	41.8	26.7	1.6 × 10^8^	1.2 × 10^8^	1.1 × 10^8^	0.97	2.7
PYCl/CB[6]	334	388	500	3.62	5.40	3.7	81.2	1.0 × 10^7^	4.2 × 10^7^	2.2 × 10^8^	150	34.8
PIC/CB[6]	407	477	575	1.53	1.40	2.6	3.9	1.7 × 10^7^	6.1 × 10^8^	2.5 × 10^7^	27.9	6.9 × 10^2^
PEC/CB[6]	429	494	—^*f*^	7.52	—^*f*^	4.34	—^*f*^	5.8 × 10^6^	1.3 × 10^8^	—^*g*^	—^*g*^	—^*g*^
PC/CB[6]	250	494	—^*f*^	23.14	—^*f*^	2.60	—^*f*^	1.1 × 10^6^	4.2 × 10^7^	—^*g*^	—^*g*^	—^*g*^

^*a*^The radiative decay rate constant of fluorescence *K*Fluor = *Φ*_Fluo_/*τ*_Fluo_.

^*b*^The nonradiative decay rate constant of fluorescence *K*Fluonr = (1 – *Φ*_Fluo_ – *Φ*_Phos_)/*τ*_Fluo_.

^*c*^The intersystem crossing rate constant *K*_isc_ = *Φ*_Phos_/*τ*_Fluo_.

^*d*^The radiative decay rate constant of phosphorescence *K*Phosr = *Φ*_Phos_/*τ*_Phos_.

^*e*^The nonradiative decay rate constant of phosphorescence *K*Phosnr = (1 – *Φ*_Phos_)/*τ*_Phos_.

^*f*^Not detected.

^*g*^Not calculated.

**Fig. 2:**
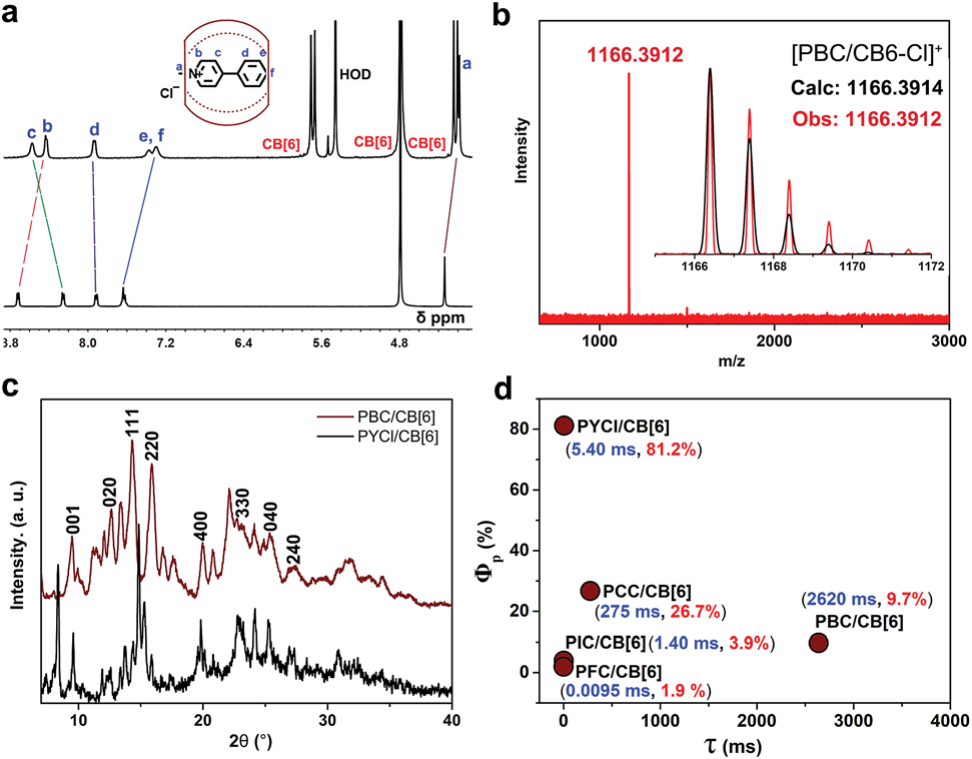
(a) ^1^H NMR spectra of PBC/CB[6] and PBC; (b) high resolution mass spectrometry of PBC/CB[6]; (c) XRD patterns of PBC/CB[6] (wine) and PYCl/CB[6] (black); (d) phosphorescence quantum yield (*Φ*_p_) and lifetime (*τ*) of PX/CB[6].

The complex was further characterized by ^1^H NMR, which revealed that methyl protons *H*_a_ and the aromatic protons *H*_b_, and *H*_e,f_ of PBC underwent enormous upfield shifts of 0.15, 0.29 and 0.26 ppm, respectively (Fig. 2a). These complexation-induced shifts indicated that PBC was deeply encapsulated into the cavity of CB[6]. High resolution mass spectrometry also proved the formation of the PBC/CB[6] complex (Fig. 2b). The intense peak of [M–Cl]^+^ (*m*/*z* 1166.3912) fitted well with the calculated value (1166.3914). The powder X-ray diffraction (XRD) pattern of the PBC/CB[6] complex revealed its ordered array like that of PYCl/CB[6] (Fig. 2c). The XRD peaks with *d*-spacings of 5.58 and 3.84 Å were indexed to diffractions from the (220) and (330) planes, respectively. The *d*-spacings of 7.00, 4.44, 3.51, and 3.27 Å were assigned to (020), (400), (040), and (240) planes (Fig. 2c). Taking into account the extremely similar structures of PBC and PYCl, this result was reasonable. To verify if the arrangement of the complex influences the lifetime, we further ground the complex powder. The XRD patterns revealed that many peaks became weaker and some even disappeared, indicating the loss of order (Fig. S18a†). But the nearly identical lifetimes (2.61 s and 2.62 s) confirmed that the change in the microstructure had little effect on the phosphorescence of the complex, which further proved the aforementioned proposal that each complex was an individual phosphor and the phosphorescence originated from complexation (Fig. S18b†).

We then synthesized guests with halogen atoms (F, Cl and I) possessing different coupling abilities to explore the relationship between the structure of the guests and the phosphorescence properties of the complexes. Besides, methyl-pyridinium chloride (PC) and styryl-methyl-pyridinium chloride (PEC) were also synthesized to verify whether the structure of phenylmethylpyridinium was necessary for generating phosphorescence (Scheme 1). Interestingly, the PFC/CB[6], PCC/CB[6] and PIC/CB[6] complexes emitted phosphorescence peaks at 520 nm (*τ* = 0.0095 ms), 500 nm (*τ* = 275 ms) and 575 nm (*τ* = 1.40 ms), accompanied by strong fluorescence emission peaks at 434 nm (*τ* = 5.61 ns), 420 nm (*τ* = 2.52 ns) and 477 nm (*τ* = 1.53 ns) in the solid state (Table 1 and Fig. S19–S21†). In contrast, guests only showed fluorescence peaks at 443 nm (*τ* = 10.62 ns) for PFC, 434 nm (*τ* = 3.49 ns) for PCC and 505 nm (*τ* = 1.20 ns) for PIC (Fig. S22†). These results proved the universality of the solid-state supramolecular strategy and indicated that the phosphorescence properties of the complex could be tuned by substituents. When the aromatic moieties were separated (PEC) or broken (PC), no phosphorescence occurred in the monomer (PEC and PC) or the complex (PEC/CB[6] and PC/CB[6]) (Fig. S23 and S24†). This means that the structure of (substituted) phenylmethylpyridinium was indispensable for generating phosphorescence of the complex in these cases. The high resolution mass spectra showed an intense peak of [PX/CB6-Cl]^+^, which proved the complexation between these guests and CB[6] (Fig. S25†). Moreover, enormous upfield shifts of methyl protons and aromatic protons of guests further confirmed that these guests were deeply complexed by CB[6] (Fig. S26–S30†).

On the basis of the measured quantum yields and lifetimes of these complexes, the radiative and nonradiative decay rate constants could be calculated following the standard methods (Table 1).^28^,^40^–^42^ Eqn (1) and (2) in Scheme 1 indicated that small *K*Phosr + *K*Phosnr was beneficial for achieving long lifetime, while high *Φ*_Phos_ was the comprehensive result of efficient ISC (high *Φ*_isc_) and high efficiency of phosphorescence (*K*Phosr/(*K*Phosr + *K*Phosnr)). The intersystem crossing rate constant *K*_isc_ of complexes ranged between 1.2 × 10^7^ and 2.2 × 10^8^ s^–1^. The fast ISC of S_1_ → T_*n*_ (*n* ≥ 1) (1.1 × 10^8^ s^–1^ for PCC/CB[6] and 2.2 × 10^8^ s^–1^ for PYCl/CB[6]) produced a sufficient population of T_1_ and therefore provided one of the essential conditions for efficient phosphorescence. The slow nonradiative decay (*K*Phosnr = 2.7 s^–1^ for PCC/CB[6] and 34.8 s^–1^ for PYCl/CB[6]) of phosphorescence provided another one, resulting in high phosphorescence quantum yield (81.2% for PYCl/CB[6] and 26.7% for PCC/CB[6]). For PBC/CB[6], the extremely low value of *K*Phosr + *K*Phosnr (0.377 s^–1^) made T_1_ deactivation a very slow process and therefore resulted in ultralong lifetime. The *K*Phosr + *K*Phosnr of PCC/CB[6] was also small enough (3.67 s^–1^) to produce an afterglow (Fig. 1c and Video 2†). Moreover, there was no positive correlation between the phosphorescence quantum yield (*Φ*_Phos_) of the complexes and the atomic number of the substituted halogen (Table 1 and Fig. S31–S33†). A possible explanation was proposed: according to eqn (2) in Scheme 1, the increase of atomic number (from F to I) would improve *Φ*_isc_ and *K*Phosr, which was beneficial for achieving high *Φ*_Phos_.^6^ But *K*Phosnr would be improved too and *Φ*_Phos_ would decrease. Therefore, *K*Phosr/(*K*Phosr + *K*Phosnr) would increase or decrease, making *Φ*_Phos_ either higher or lower.

**Fig. 3:**
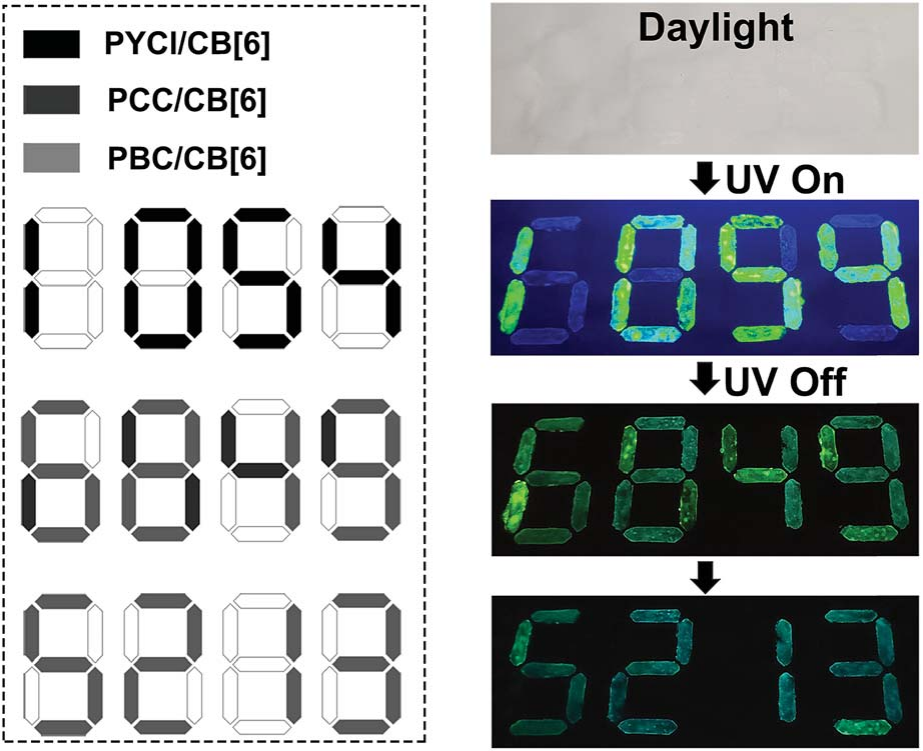
Schematic illustration of lifetime-encoding for security applications using PYCl/CB[6], PCC/CB[6] and PBC/CB[6].

Information encryption and anti-counterfeiting are of great significance and have received enormous attention in this information era.^43^–^49^ On the basis of distinct lifetime and robust phosphorescence properties of these complexes, a triple encoding model was fabricated. As illustrated in Fig. 3 (dotted box), the digital pattern was painted with different complexes on flexible paper, among which PYCl/CB[6] was used to label the black part, the dark gray part was coated with PCC/CB[6], and the light gray part was painted with PBC/CB[6]. In daylight, only a colorless pattern was obtained. When a UV lamp with 365 nm excitation was turned on, an intense green “1054” appeared because of the high quantum yield of PYCl/CB[6] (Fig. 3 and Video 3†). On switching the UV lamp off, the relatively short lifetime of PYCl/CB[6] (5.40 ms) made its phosphorescence disappear immediately, while long-lived PCC/CB[6] (275 ms) and PBC/CB[6] (2620 ms) contributed to the moderate green “6849”. Later, the phosphorescence of PCC/CB[6] became invisible, and the pattern that remained was “5213”. Therefore, we achieved triple lifetime-encoding for information encryption and anti-counterfeiting by subtle utilization of the diverse lifetimes of phosphorescent complexes.

## Conclusion

In conclusion, we present a concise and universal solid-state supramolecular strategy to realize purely organic heavy-atom-free ultralong RTP based on CB[6] and phenylmethylpyridinium. The strategy involving the enhancement of ISC and suppression of nonradiative decay through the complexation of CB[6] can remarkably promote RTP of phenylmethylpyridinium and its derivatives under ambient conditions. Significantly, PBC/CB[6] powder exhibits a phosphorescence lifetime of 2.62 s and a decent efficiency of 9.7%. Moreover, the structure of phenylmethylpyridinium (PBC, PFC, PCC, PYCl and PIC) is vital for the generation of phosphorescence. Notably, PBC/CB[6], PCC/CB[6] and PYCl/CB[6] were successfully applied in triple lifetime-encoding by utilizing their different lifetimes, showing great potential for application in information encryption and anti-counterfeiting. This strategy possesses the advantages of facile preparation, universality and robust RTP without rigorous conditions such as low temperature, crystallization and deoxygenation. The result will help to understand purely organic ultralong RTP and promote the generation of better (longer lifetime, higher efficiency and more robust) RTP materials that may stimulate the development of data encryption, organic devices, bio-imaging, and so on.

## Conflicts of interest

There are no conflicts to declare.

## Supplementary Material

Supplementary information

Supplementary movie

Supplementary movie

Supplementary movie

## References

[cit1] Zhao Q., Huang C., Li F. (2011). Chem. Soc. Rev..

[cit2] Zhang G., Palmer G. M., Dewhirst M. W., Fraser C. L. (2009). Nat. Mater..

[cit3] Yang X., Zhou G., Wong W.-Y. (2015). Chem. Soc. Rev..

[cit4] Cai S., Shi H., Li J., Gu L., Ni Y., Cheng Z., Wang S., Xiong W.-w., Li L., An Z., Huang W. (2017). Adv. Mater..

[cit5] Xu S., Chen R., Zheng C., Huang W. (2016). Adv. Mater..

[cit6] Baroncini M., Bergamini G., Ceroni P. (2017). Chem. Commun..

[cit7] Kabe R., Adachi C. (2017). Nature.

[cit8] Gong Y., Chen G., Peng Q., Yuan W. Z., Xie Y., Li S., Zhang Y., Tang B. Z. (2015). Adv. Mater..

[cit9] Zhou B., Yan D. (2019). Adv. Funct. Mater..

[cit10] Yang J., Zhen X., Wang B., Gao X., Ren Z., Wang J., Xie Y., Li J., Peng Q., Pu K., Li Z. (2018). Nat. Commun..

[cit11] Yang Z., Mao Z., Zhang X., Ou D., Mu Y., Zhang Y., Zhao C., Liu S., Chi Z., Xu J., Wu Y.-C., Lu P.-Y., Lien A., Bryce M. R. (2016). Angew. Chem., Int. Ed..

[cit12] Zhang T., Zhao Z., Ma H., Zhang Y., Yuan W. Z. (2019). Chem.–Asian J..

[cit13] Mao Z., Yang Z., Fan Z., Ubba E., Li W., Li Y., Zhao J., Yang Z., Aldred M. P., Chi Z. (2019). Chem. Sci..

[cit14] Chen G., Feng H., Feng F., Xu P., Xu J., Pan S., Qian Z. (2018). J. Phys. Chem. Lett..

[cit15] He Z., Zhao W., Lam J. W. Y., Peng Q., Ma H., Liang G., Shuai Z., Tang B. Z. (2017). Nat. Commun..

[cit16] Bolton O., Lee K., Kim H.-J., Lin K. Y., Kim J. (2011). Nat. Chem..

[cit17] He Z., Gao H., Zhang S., Zheng S., Wang Y., Zhao Z., Ding D., Yang B., Zhang Y., Yuan W. Z. (2019). Adv. Mater..

[cit18] Ogoshi T., Tsuchida H., Kakuta T., Yamagishi T.-a., Taema A., Ono T., Sugimoto M., Mizuno M. (2018). Adv. Funct. Mater..

[cit19] Kwon M. S., Yu Y., Coburn C., Phillips A. W., Chung K., Shanker A., Jung J., Kim G., Pipe K., Forrest S. R., Youk J. H., Gierschner J., Kim J. (2015). Nat. Commun..

[cit20] Gan N., Shi H., An Z., Huang W. (2018). Adv. Funct. Mater..

[cit21] Parke S. M., Hupf E., Matharu G. K., de Aguiar I., Xu L., Yu H., Boone M. P., de Souza G. L. C., McDonald R., Ferguson M. J., He G., Brown A., Rivard E. (2018). Angew. Chem., Int. Ed..

[cit22] Mieno H., Kabe R., Notsuka N., Allendorf M. D., Adachi C. (2016). Adv. Opt. Mater..

[cit23] Wu H., Chi W., Chen Z., Liu G., Gu L., Bindra A. K., Yang G., Liu X., Zhao Y. (2019). Adv. Funct. Mater..

[cit24] Li Q., Zhou M., Yang M., Yang Q., Zhang Z., Shi J. (2018). Nat. Commun..

[cit25] Hirata S., Totani K., Yamashita T., Adachi C., Vacha M. (2014). Nat. Mater..

[cit26] Jiang K., Wang Y., Cai C., Lin H. (2018). Adv. Mater..

[cit27] Chen X., Xu C., Wang T., Zhou C., Du J., Wang Z., Xu H., Xie T., Bi G., Jiang J., Zhang X., Demas J. N., Trindle C. O., Luo Y., Zhang G. (2016). Angew. Chem., Int. Ed..

[cit28] An Z., Zheng C., Tao Y., Chen R., Shi H., Chen T., Wang Z., Li H., Deng R., Liu X., Huang W. (2015). Nat. Mater..

[cit29] Bian L., Shi H., Wang X., Ling K., Ma H., Li M., Cheng Z., Ma C., Cai S., Wu Q., Gan N., Xu X., An Z., Huang W. (2018). J. Am. Chem. Soc..

[cit30] Shoji Y., Ikabata Y., Wang Q., Nemoto D., Sakamoto A., Tanaka N., Seino J., Nakai H., Fukushima T. (2017). J. Am. Chem. Soc..

[cit31] Su Y., Phua S. Z. F., Li Y., Zhou X., Jana D., Liu G., Lim W. Q., Ong W. K., Yang C., Zhao Y. (2018). Sci. Adv..

[cit32] Ma X., Xu C., Wang J., Tian H. (2018). Angew. Chem., Int. Ed..

[cit33] Li D., Lu F., Wang J., Hu W., Cao X.-M., Ma X., Tian H. (2018). J. Am. Chem. Soc..

[cit34] Ma X., Wang J., Tian H. (2019). Acc. Chem. Res..

[cit35] Wang X.-F., Xiao H., Chen P.-Z., Yang Q.-Z., Chen B., Tung C.-H., Chen Y.-Z., Wu L.-Z. (2019). J. Am. Chem. Soc..

[cit36] Ma H., Peng Q., An Z., Huang W., Shuai Z. (2019). J. Am. Chem. Soc..

[cit37] Zhang Z.-Y., Chen Y., Liu Y. (2019). Angew. Chem., Int. Ed..

[cit38] Zhang Y., Zhou T.-Y., Zhang K.-D., Dai J.-L., Zhu Y.-Y., Zhao X. (2014). Chem.–Asian J..

[cit39] Lee J. W., Samal S., Selvapalam N., Kim H.-J., Kim K. (2003). Acc. Chem. Res..

[cit40] Byeon C. C., McKerns M. M., Sun W., Nordlund T. M., Lawson C. M., Gray G. M. (2004). Appl. Phys. Lett..

[cit41] Yoon S.-J., Kim J. H., Kim K. S., Chung J. W., Heinrich B., Mathevet F., Kim P., Donnio B., Attias A.-J., Kim D., Park S. Y. (2012). Adv. Funct. Mater..

[cit42] Chow P. C. Y., Albert-Seifried S., Gélinas S., Friend R. H. (2014). Adv. Mater..

[cit43] Zhou Y., Han S.-T., Chen X., Wang F., Tang Y.-B., Roy V. A. L. (2014). Nat. Commun..

[cit44] Xue J., Zhou Z.-K., Wei Z., Su R., Lai J., Li J., Li C., Zhang T., Wang X.-H. (2015). Nat. Commun..

[cit45] Huang Q., Mei X., Xie Z., Wu D., Yang S., Gong W., Chi Z., Lin Z., Ling Q. (2019). J. Mater. Chem. C.

[cit46] Wang Z., Zhu C.-Y., Yin S.-Y., Wei Z.-W., Zhang J.-H., Fan Y.-N., Jiang J.-J., Pan M., Su C.-Y. (2019). Angew. Chem., Int. Ed..

[cit47] Jiang K., Wang Y., Cai C., Lin H. (2017). Chem. Mater..

[cit48] Louis M., Thomas H., Gmelch M., Haft A., Fries F., Reineke S. (2019). Adv. Mater..

[cit49] Zhou Q., Wang Z., Dou X., Wang Y., Liu S., Zhang Y., Yuan W. Z. (2019). Mater. Chem. Front..

