# Supplementary material for: Ultralong room-temperature phosphorescence of a solid-state supramolecule between phenylmethylpyridinium and cucurbit[6]uril
Source: Chem Sci. 2019 Jul 1;10(33):7773–8. doi: 10.1039/c9sc02633a (PMC6764277; doi:10.1039/c9sc02633a)
Supplement: Supplementary file 1 [file SC-010-C9SC02633A-s001.pdf]

Electronic Supplementary Material (ESI) for Chemical Science.
This journal is © The Royal Society of Chemistry 2019

Supporting Information

Ultralong Room-Temperature Phosphorescence of Solid-State Supramolecule between Phenylmethylpyridinium and Cucurbit[6]uril

Zhi-Yuan Zhang, [†] *and Yu Liu* [*,†]

†. College of Chemistry, State Key Laboratory of Elemento-Organic Chemistry, Nankai University, Tianjin 300071, P. R. China.

Corresponding Author

*Email: yuliu@nankai.edu.cn

Synthesis of PX 3
Figures S1-14: ^{1}H NMR, ^{13}C NMR and HRMS spectra of PX........................3-10
Figure S15: Photophysical properties of PBC..10
Figure S16: Time-resolved PL decay of PBC/CB[6]......................................11
Figure S17: Quantum efficiency of PBC/CB[6]. 11
Figure S18: XRD patterns of PBC/CB[6]. 12
Figures S19-21: Photophysical properties of PX/CB[6] 12-13
Figure S22: Photophysical properties of PFC, PCC and PIC...........................14
Figure S23: Photophysical properties of PEC/CB[6] and PC/CB[6]...............15
Figure S24: Photophysical properties of PEC and PC 15
Figure S25: High resolution MALDI-TOF MS of PX/CB[6]..........................16
Figures S26-30: ^{1}H NMR spectra of PX and PX/CB[6]. 17-19
Figures S31-33: Quantum efficiency of PX/CB[6]..19-20

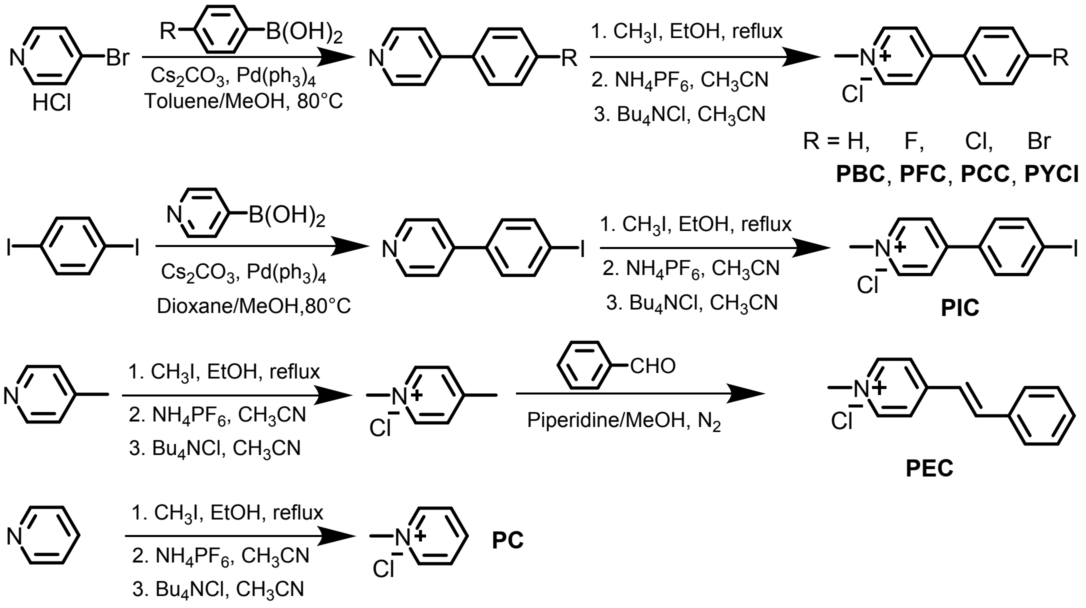

Synthesis of PX

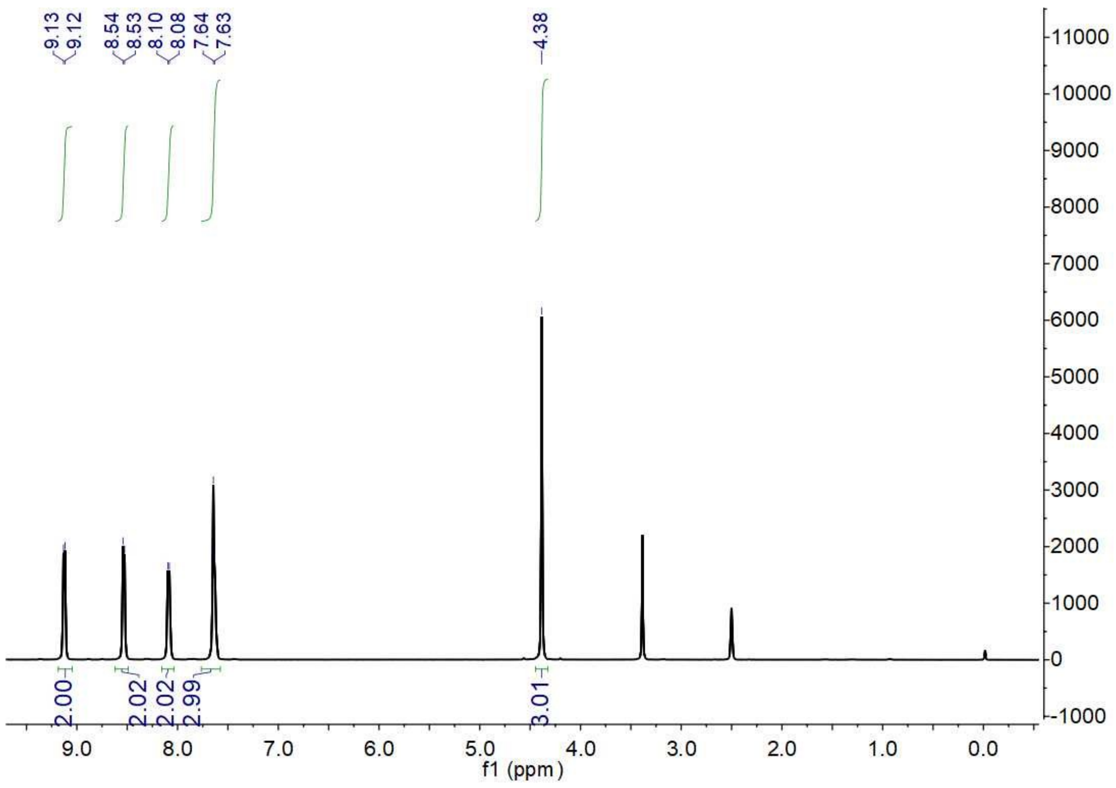

Figure S1. ^{1}H NMR (400 MHz, DMSO-d_6, 298 K) spectrum of PBC (3.0 mM).

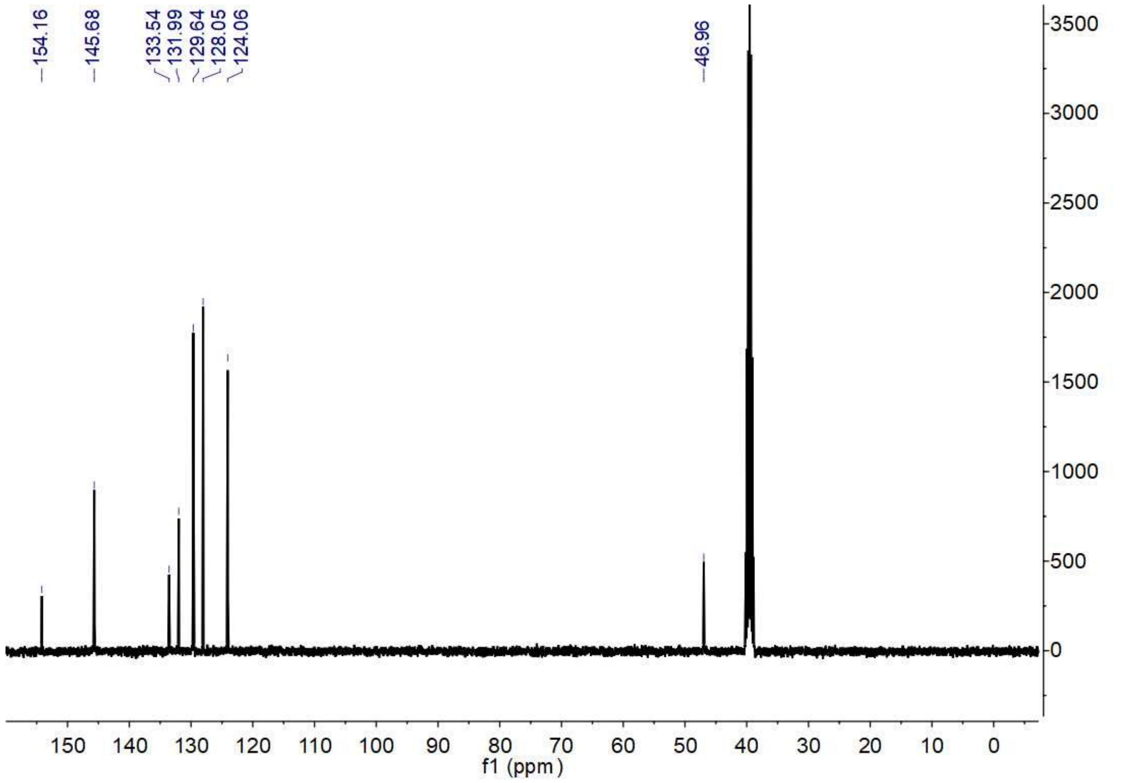

Figure S2. ^{13}C NMR (101 MHz, DMSO-d_6, 298 K) spectrum of PBC (3.0 mM).

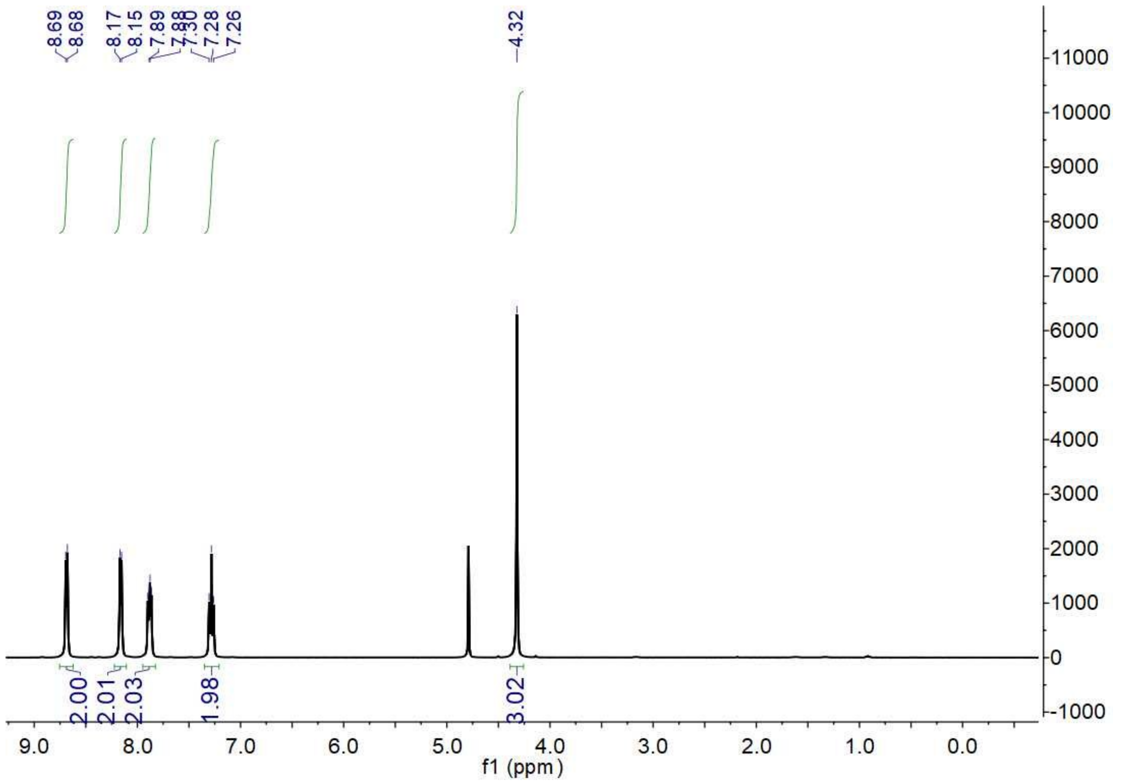

Figure S3. ^{1}H NMR (400 MHz, D_2O, 298 K) spectrum of PFC (3.0 mM).

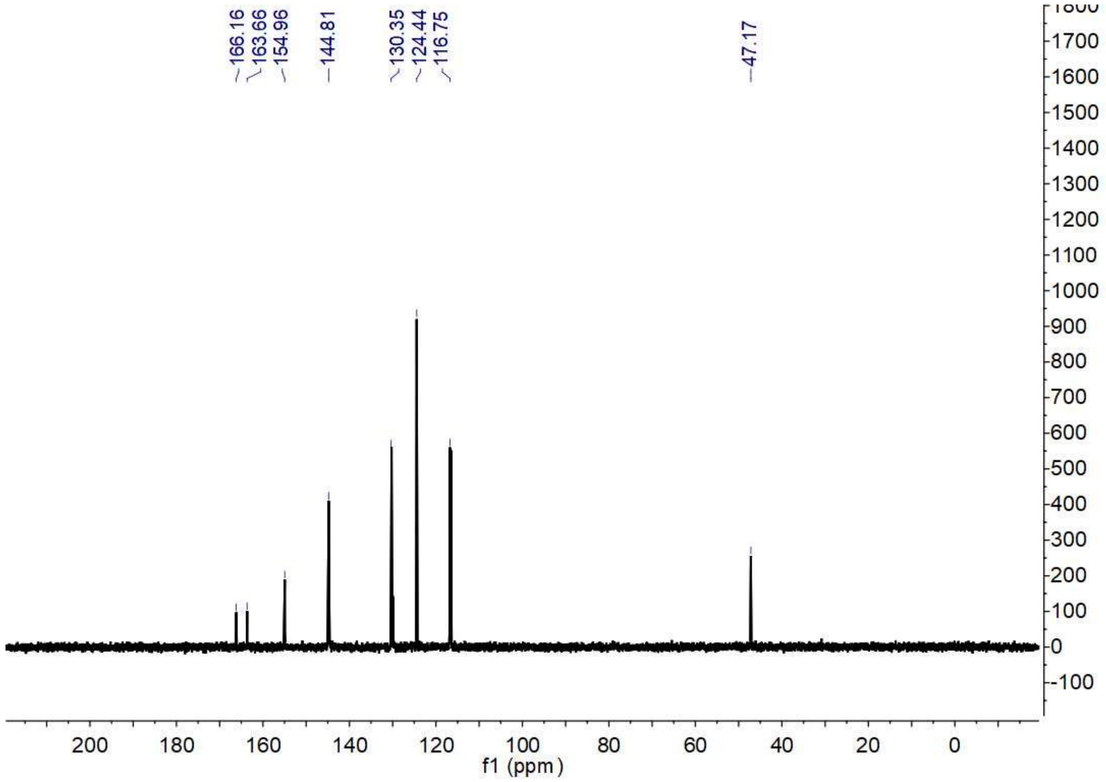

Figure S4. ^{13}C NMR (101 MHz, D_2O, 298 K) spectrum of PFC (3.0 mM).

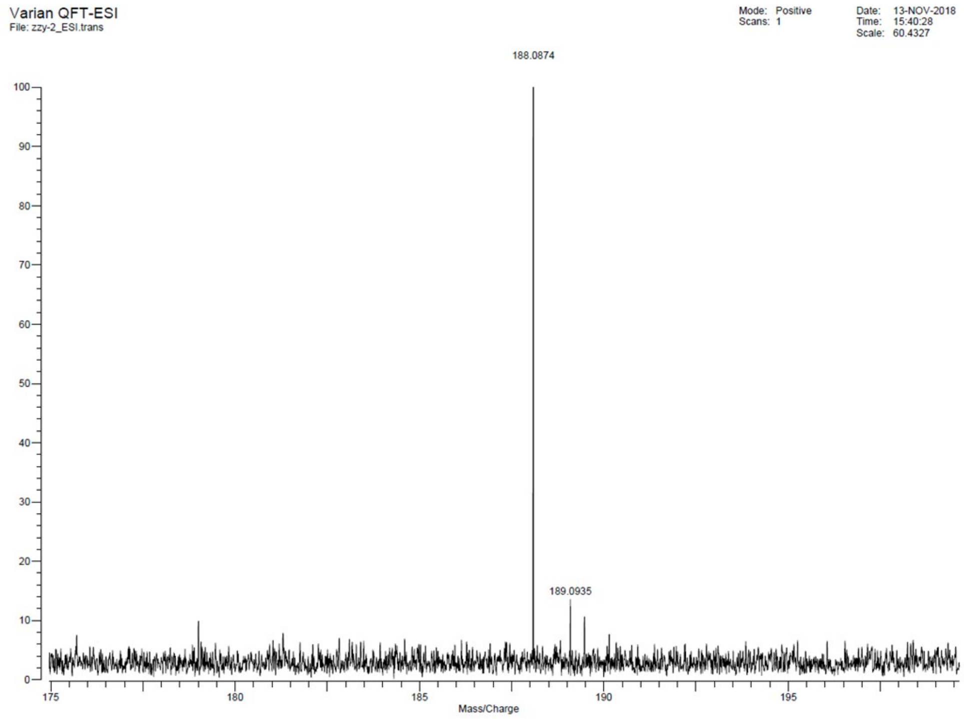

Figure S5. HRMS (ESI) spectrum of PFC.

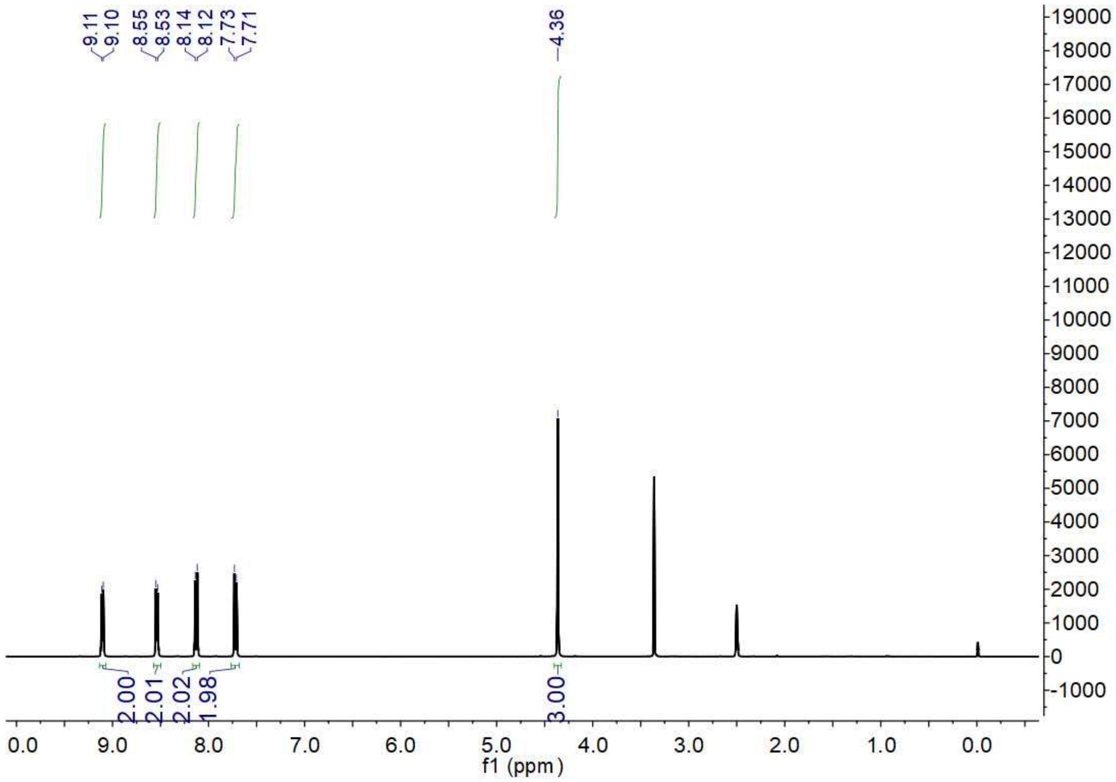

Figure S6. ^{1}H NMR (400 MHz, DMSO-d_6, 298 K) spectrum of PCC (3.0 mM).

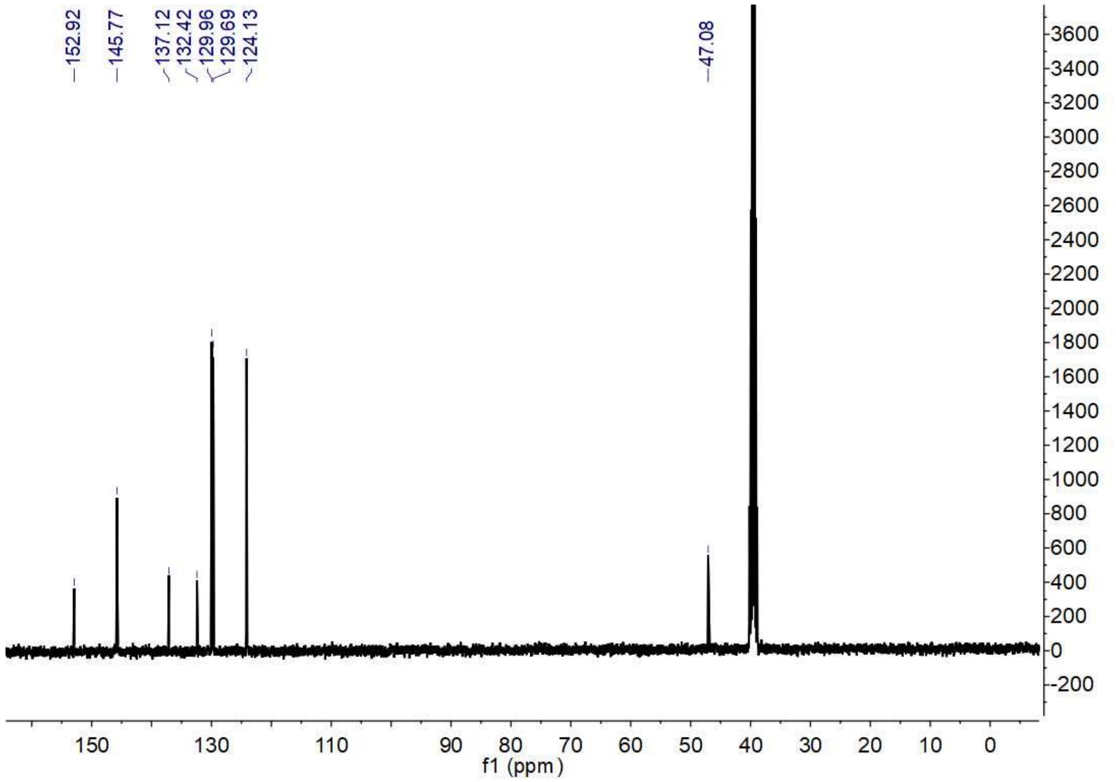

Figure S7. ^{13}C NMR (101 MHz, DMSO-d_6, 298 K) spectrum of PCC (3.0 mM).

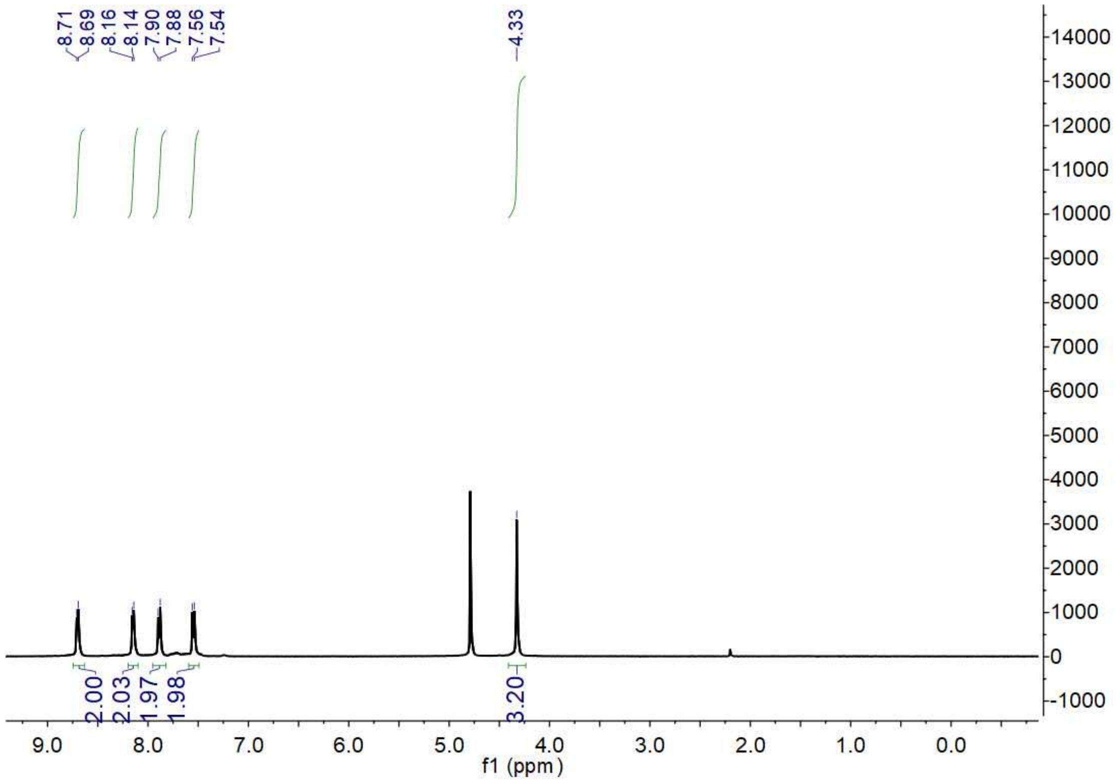

Figure S8. ^{1}H NMR (400 MHz, D_2O, 298 K) spectrum of PIC (3.0 mM).

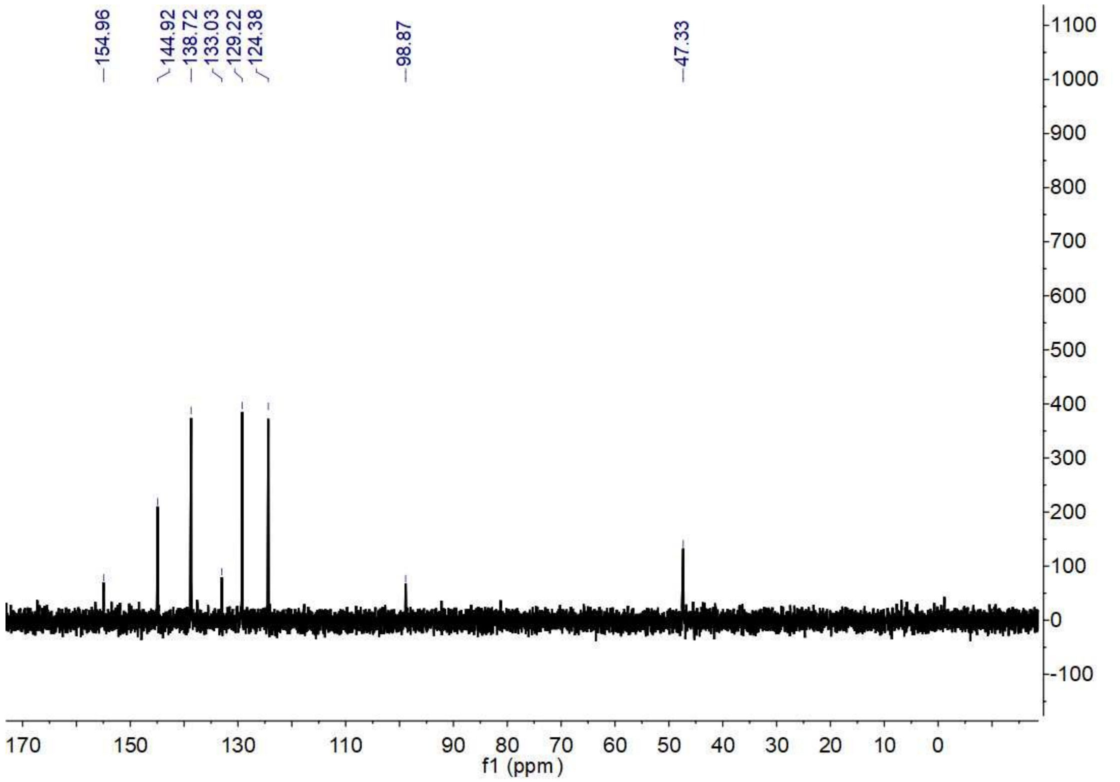

Figure S9. ^{13}C NMR (101 MHz, D_2O, 298 K) spectrum of PIC (3.0 mM).

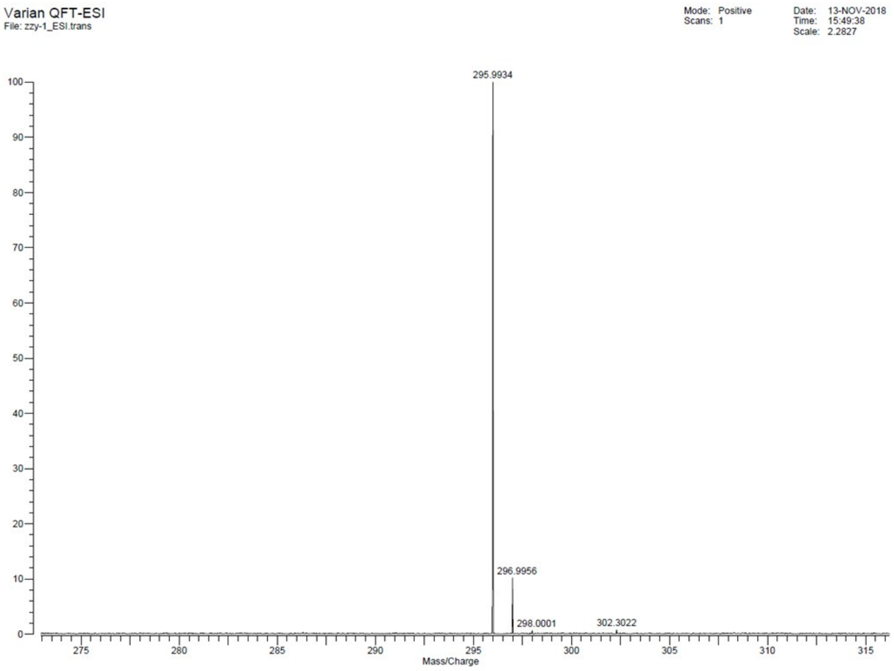

Figure S10. HRMS (ESI) spectrum of PIC.

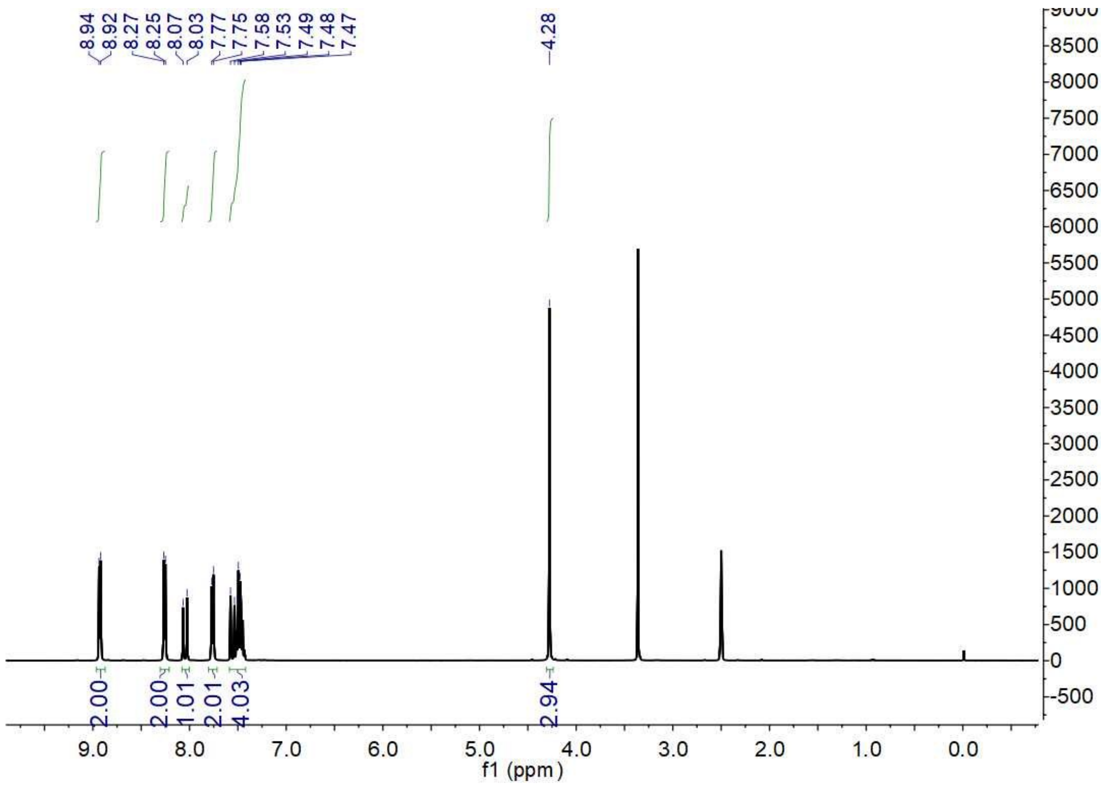

Figure S11. ^{1}H NMR (400 MHz, DMSO-d_6, 298 K) spectrum of PEC (3.0 mM).

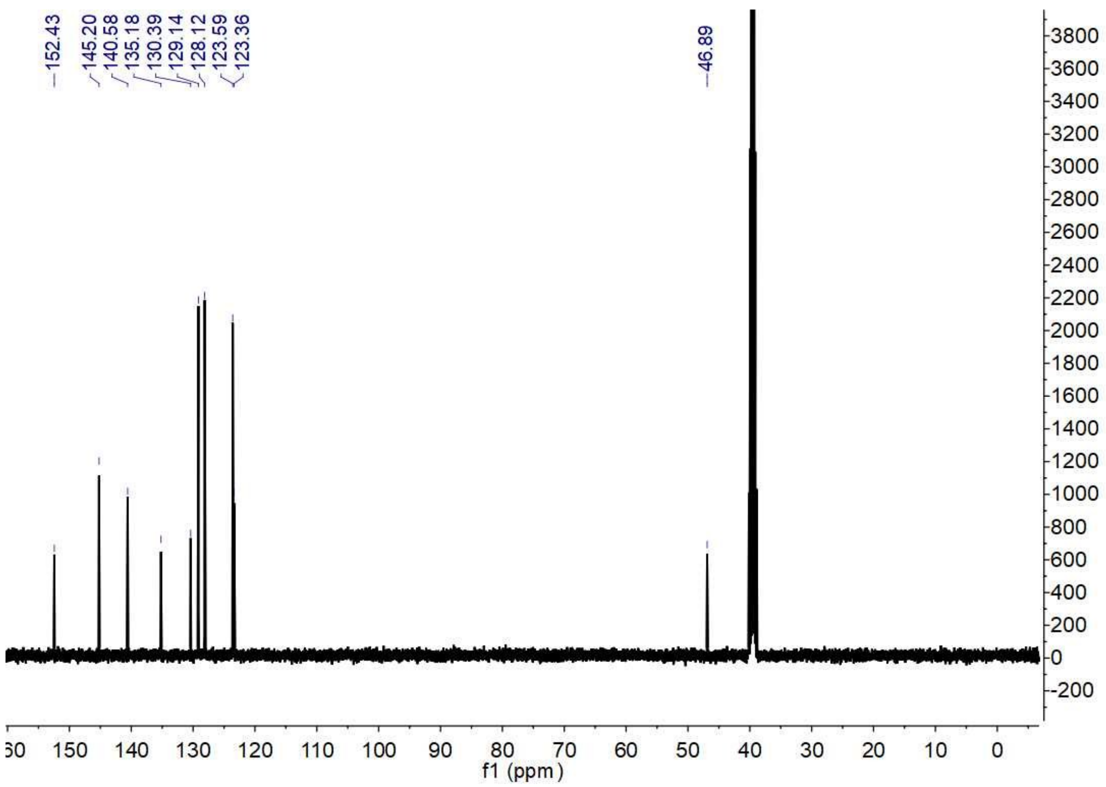

Figure S12. ^{13}C NMR (101 MHz, DMSO-d_6, 298 K) spectrum of PEC (3.0 mM).

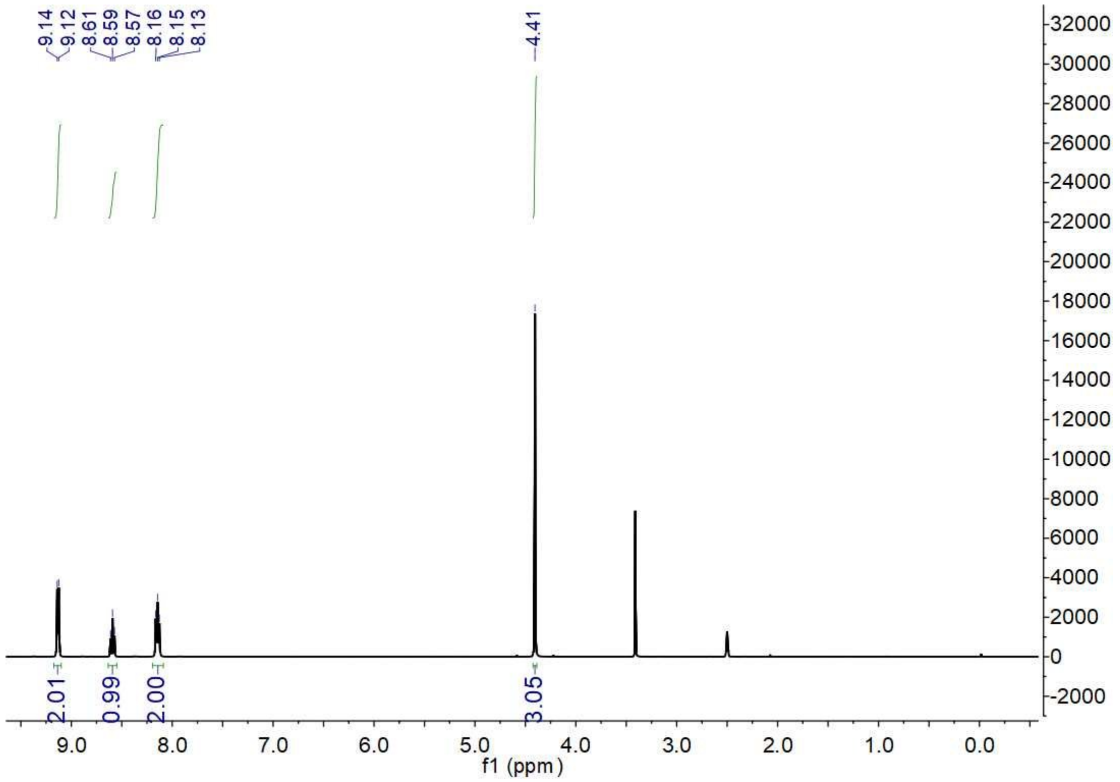

Figure S13. ^{1}H NMR (400 MHz, DMSO-d_6, 298 K) spectrum of PC (3.0 mM).

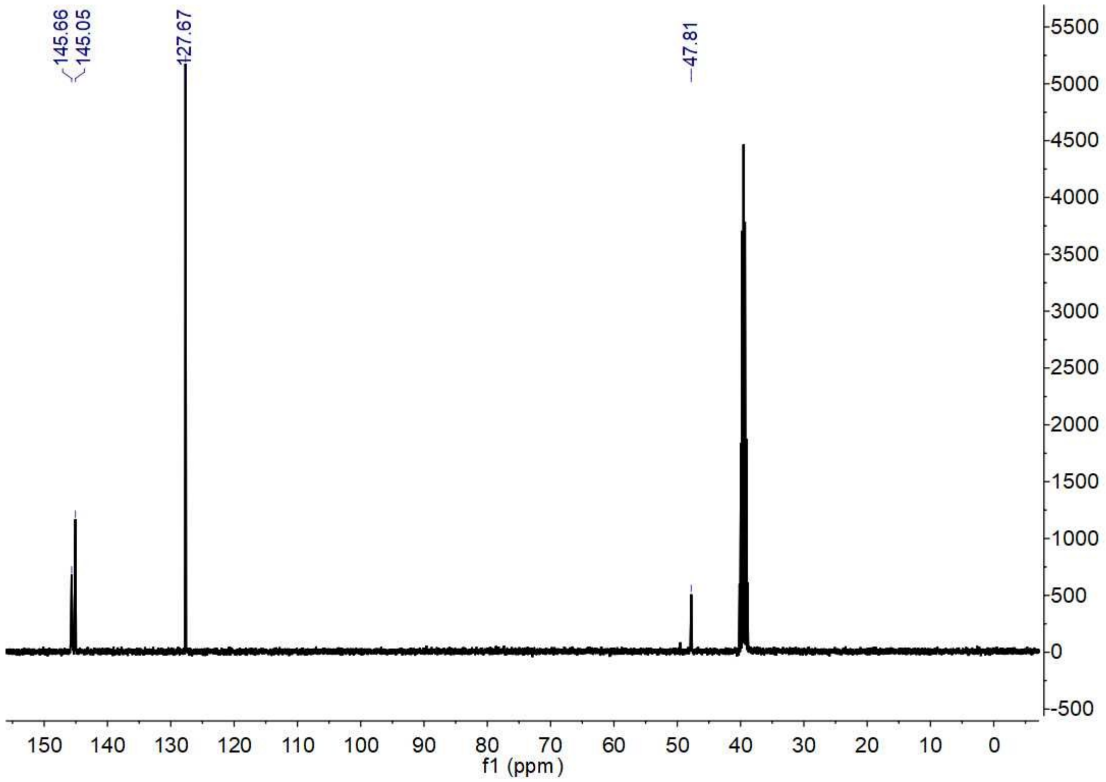

Figure S14. ^{13}C NMR (101 MHz, DMSO-d_6, 298 K) spectrum of PC (3.0 mM).

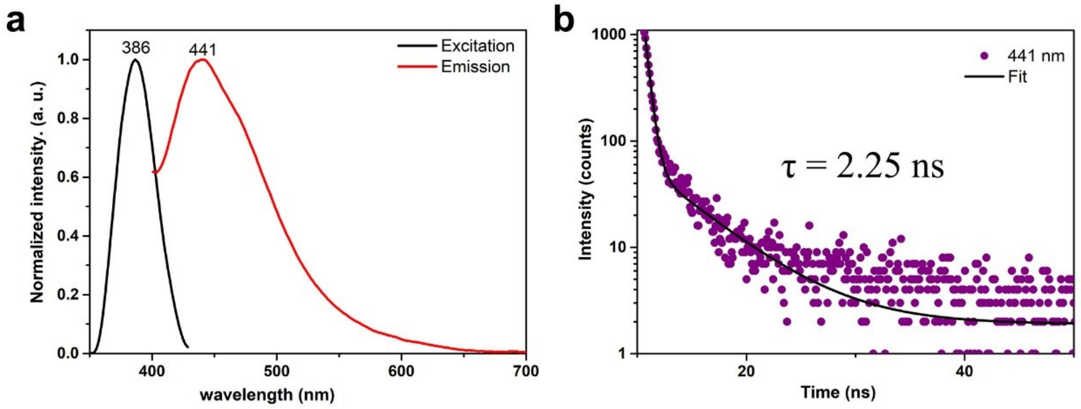

Figure S15. (a) Excitation and photoluminescence spectra of PBC in solid; (b) Time-resolved PL decay of PBC in solid at room temperature (@ 441 nm);

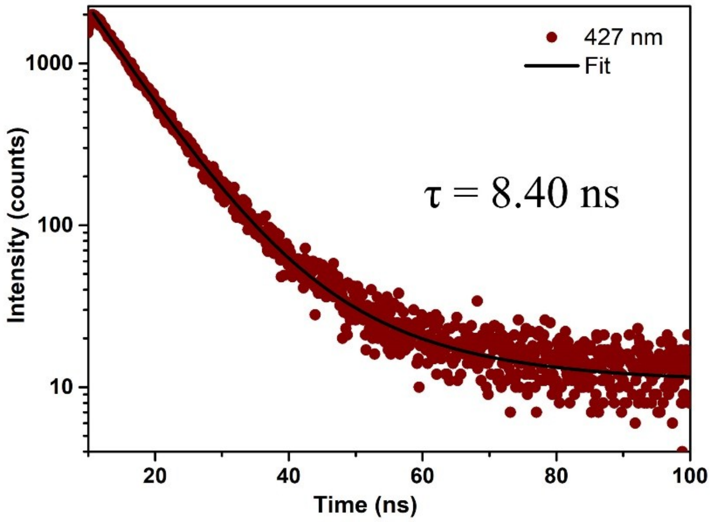

Figure S16. Time-resolved PL decay of PBC/CB[6] in solid at room temperature (@ 427 nm).

a

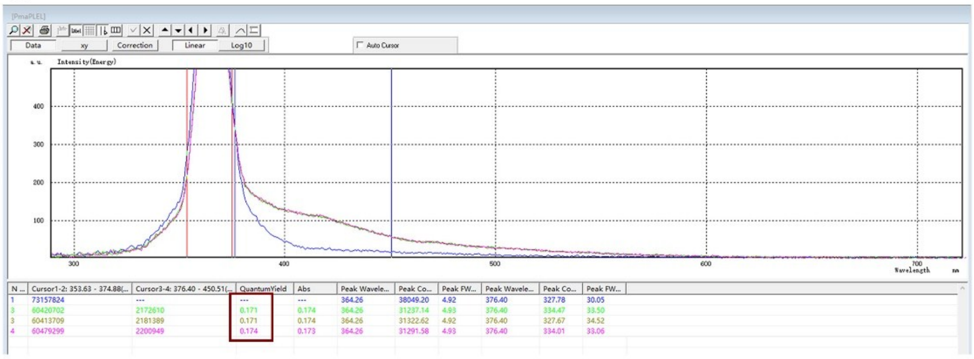

b

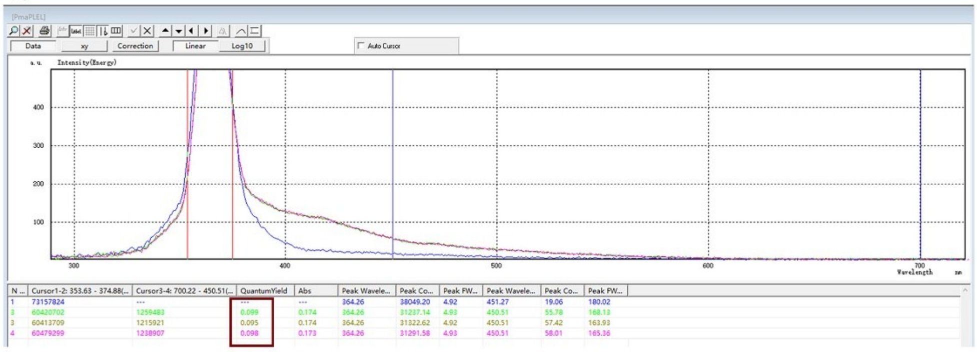

Figure S17. Fluorescence (a) and Phosphorescence (b) quantum efficiency of PBC/CB[6].

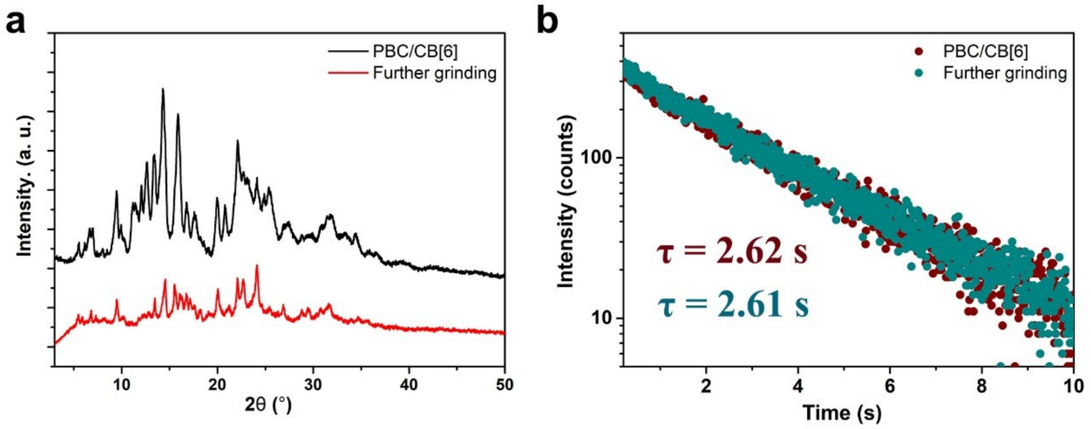

Figure S18. (a) XRD patterns of PBC/CB[6] without (black) and with (wine) further grinding; (b) Time-resolved PL decay of PBC/CB[6] without (wine) and with (cyan) further grinding.

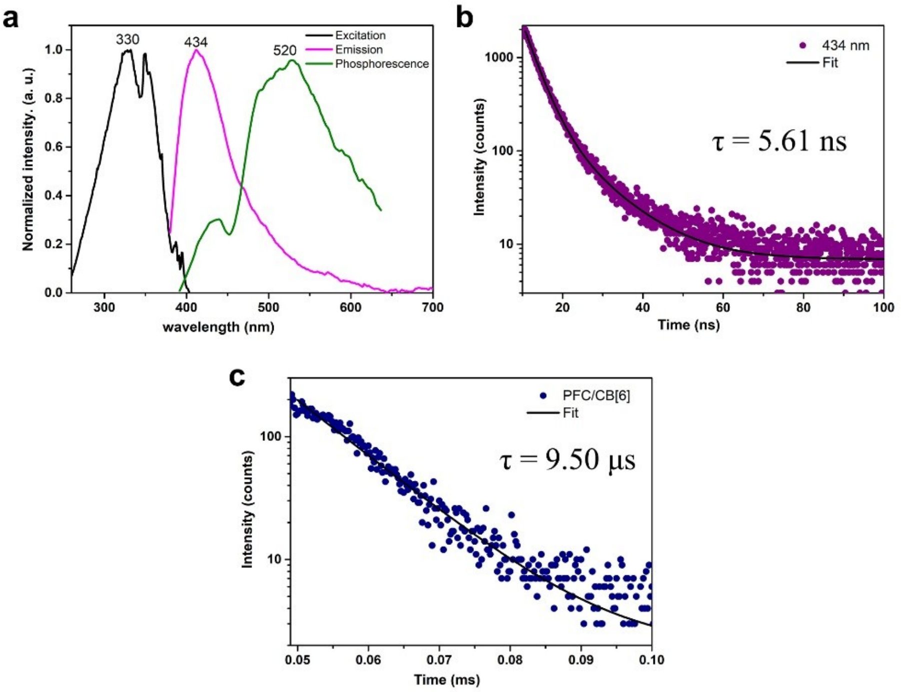

Figure S19. (a) Excitation, photoluminescence and phosphorescence spectra of PFC/CB[6] in solid; (b) Time-resolved PL decay of PFC/CB[6] in solid at room temperature (@ 434 nm); (c) Time-resolved PL decay of PFC/CB[6] in solid at room temperature (@ 520 nm).

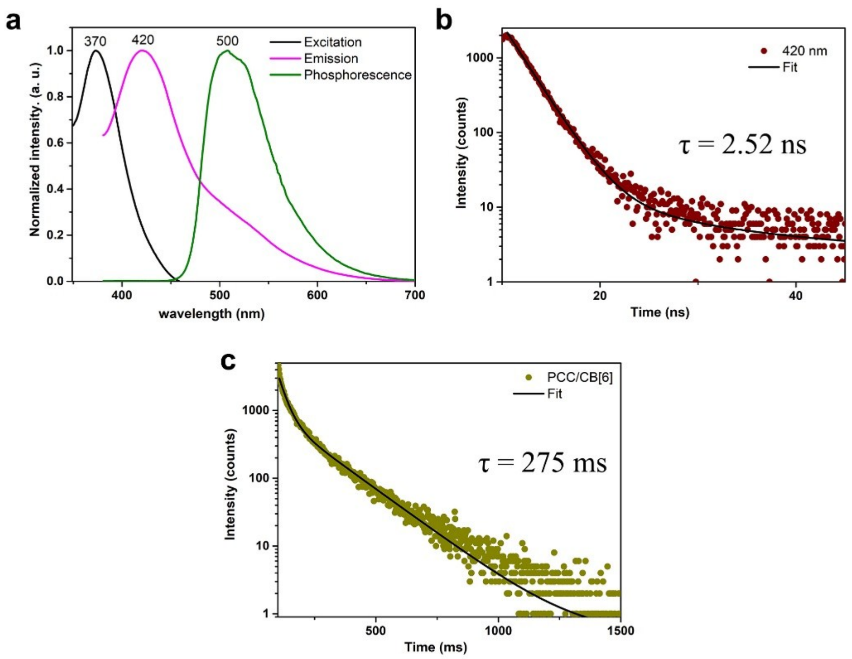

Figure S20. (a) Excitation, photoluminescence and phosphorescence spectra of PCC/CB[6] in solid; (b) Time-resolved PL decay of PCC/CB[6] in solid at room temperature (@ 420 nm); (c) Time-resolved PL decay of PCC/CB[6] in solid at room temperature (@ 500 nm).

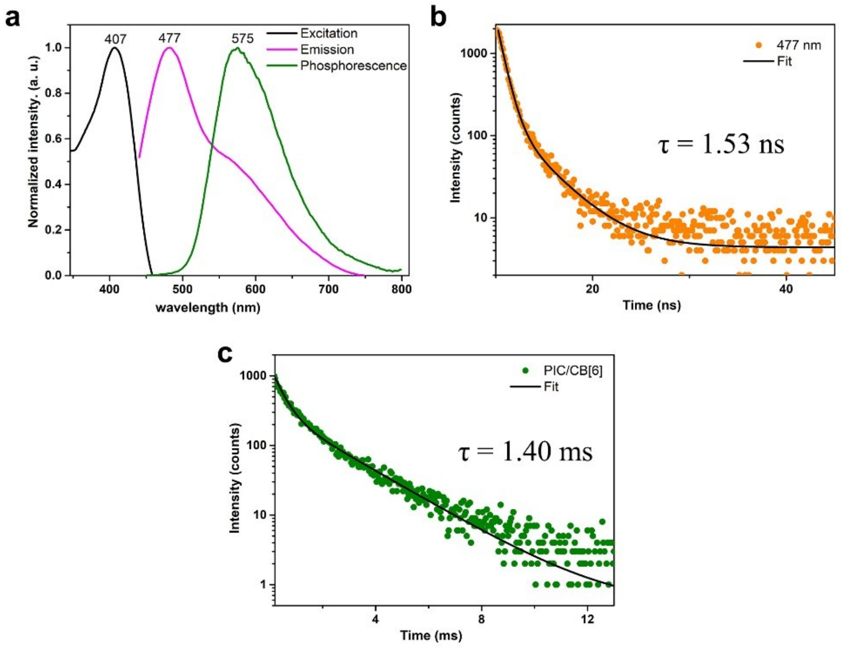

Figure S21. (a) Excitation, photoluminescence and phosphorescence spectra of PIC/CB[6] in solid; (b) Time-resolved PL decay of PIC/CB[6] in solid at room temperature (@ 477 nm); (c) Time-resolved PL decay of PIC/CB[6] in solid at room temperature (@ 575 nm).

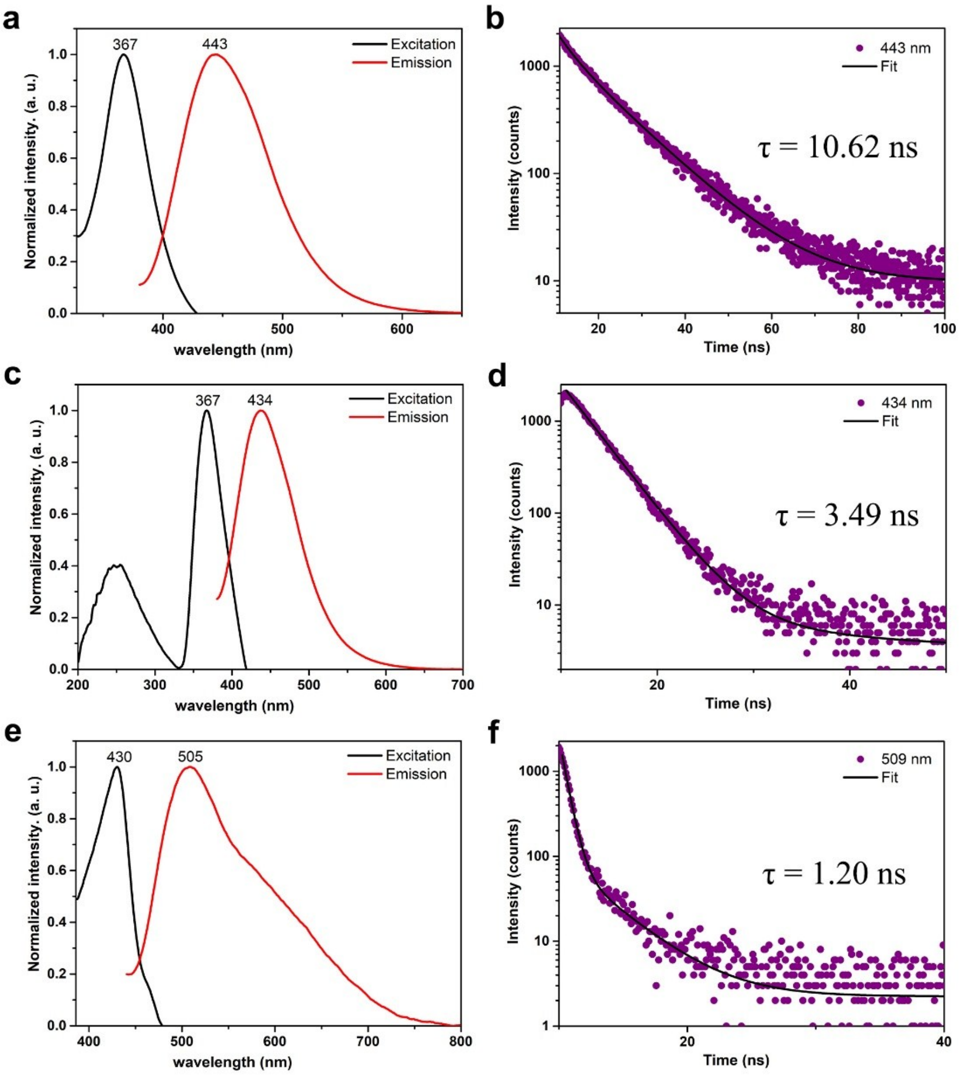

Figure S22. (a) Excitation and photoluminescence spectra of PFC in solid; (b) Time-resolved PL decay of PFC in solid at room temperature (@ 443 nm); (c) Excitation and photoluminescence spectra of PCC in solid; (d) Time-resolved PL decay of PCC in solid at room temperature (@ 434 nm); (e) Excitation and photoluminescence spectra of PIC in solid; (f) Time-resolved PL decay of PIC in solid at room temperature (@ 505 nm).

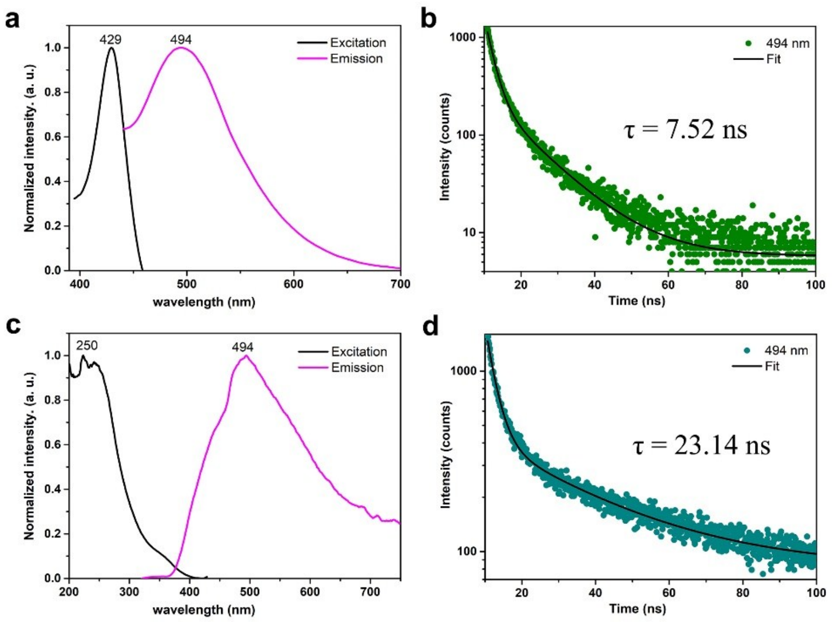

Figure S23. (a) Excitation and photoluminescence spectra of PEC/CB[6] in solid; (b) Time-resolved PL decay of PEC/CB[6] in solid at room temperature (@ 494 nm); (c) Excitation and photoluminescence spectra of PC/CB[6] in solid; (d) Time-resolved PL decay of PC/CB[6] in solid at room temperature (@ 494 nm).

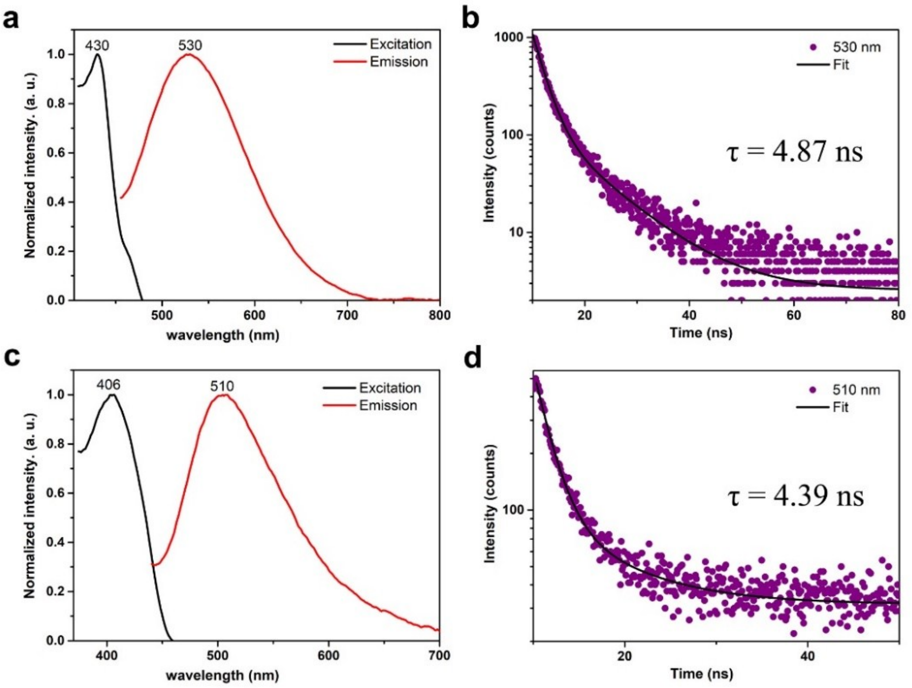

Figure S24. (a) Excitation and photoluminescence spectra of PEC in solid; (b) Time-resolved PL decay of PEC in solid at room temperature (@ 530 nm); (c) Excitation and photoluminescence spectra of PC in solid; (d) Time-resolved PL decay of PC in solid at room temperature (@ 510 nm).

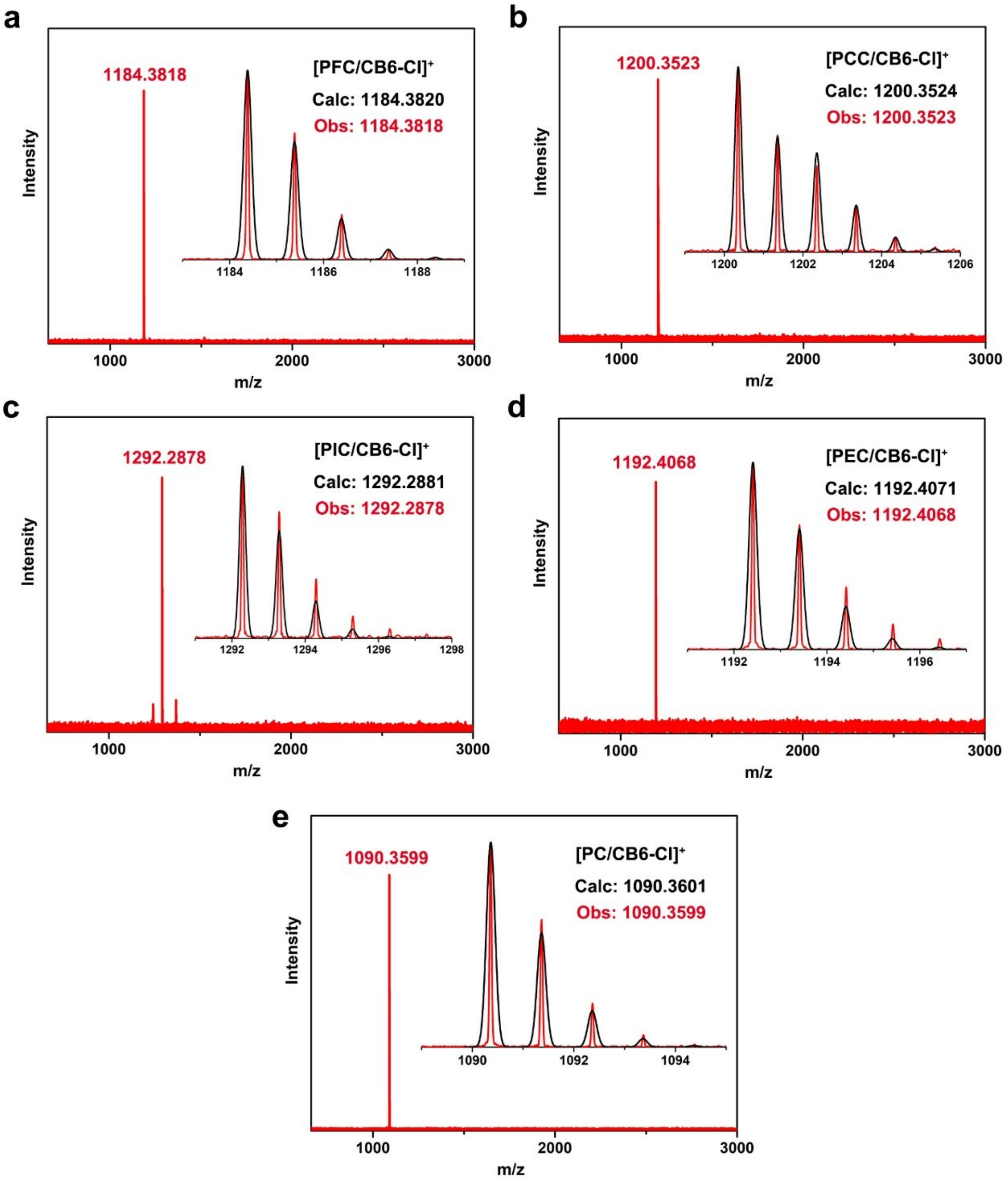

Figure S25. High resolution matrix-assisted laser desorption-ionization time of flight mass spectrometry (MALDI-TOF MS) of PX/CB[6] complexes. (a) PFC/CB[6]; (b) PCC/CB[6]; (c) PIC/CB[6]; (d) PEC/CB[6]; (e) PC/CB[6]; (Inset: zoomed peak; black: calculated data; red: observed data).

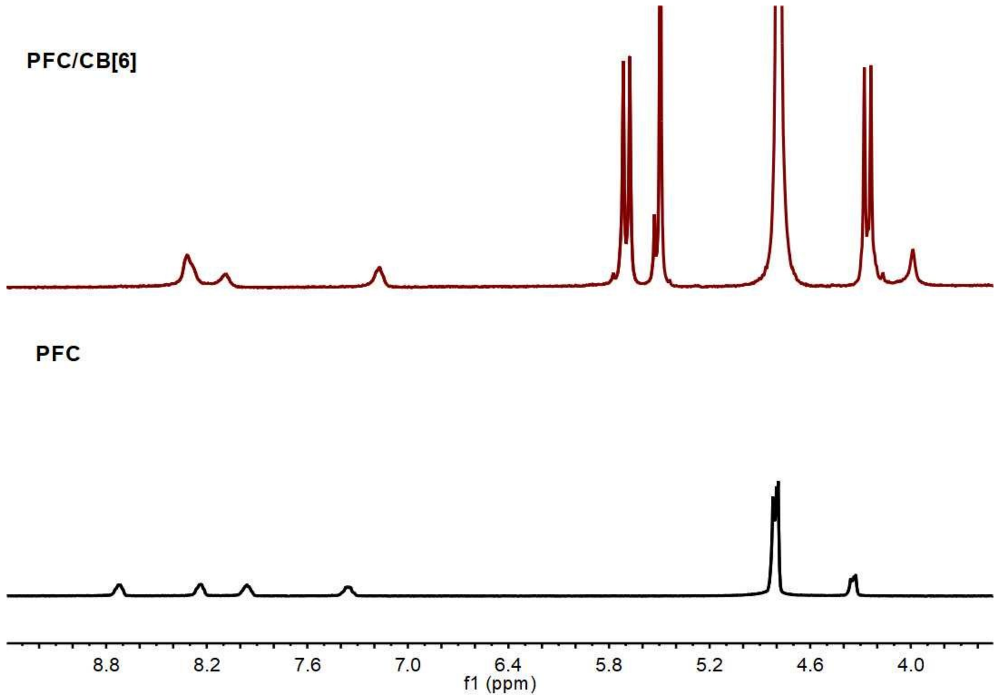

Figure S26. ^{1}H NMR (400 MHz, D_2O, 298 K) spectra of PFC/CB[6] (maroon) and PFC (black).

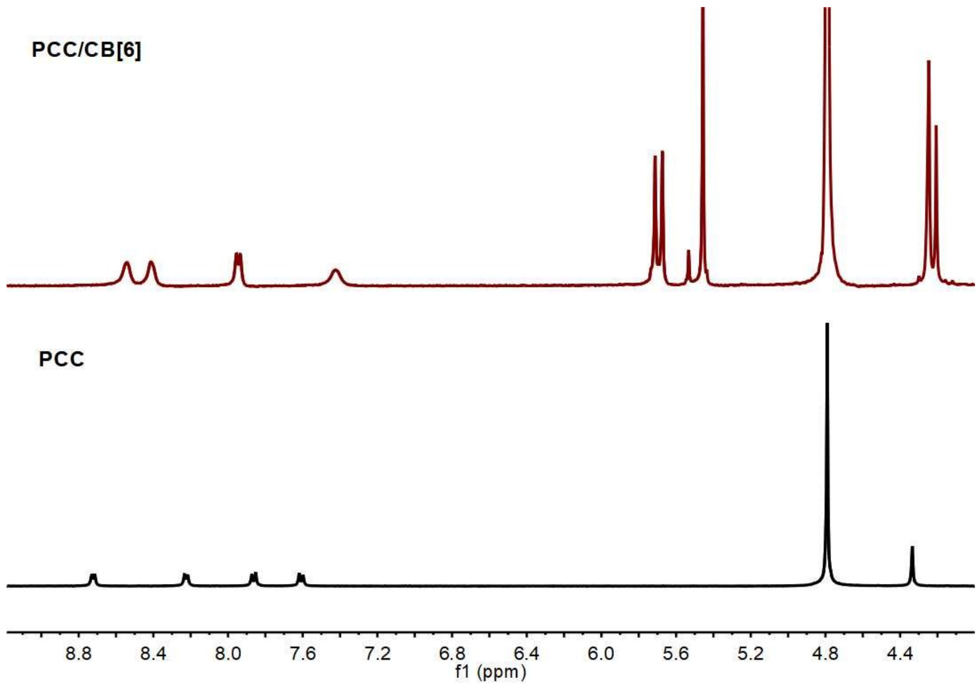

Figure S27. ^{1}H NMR (400 MHz, D_2O, 298 K) spectra of PCC/CB[6] (maroon) and PCC (black).

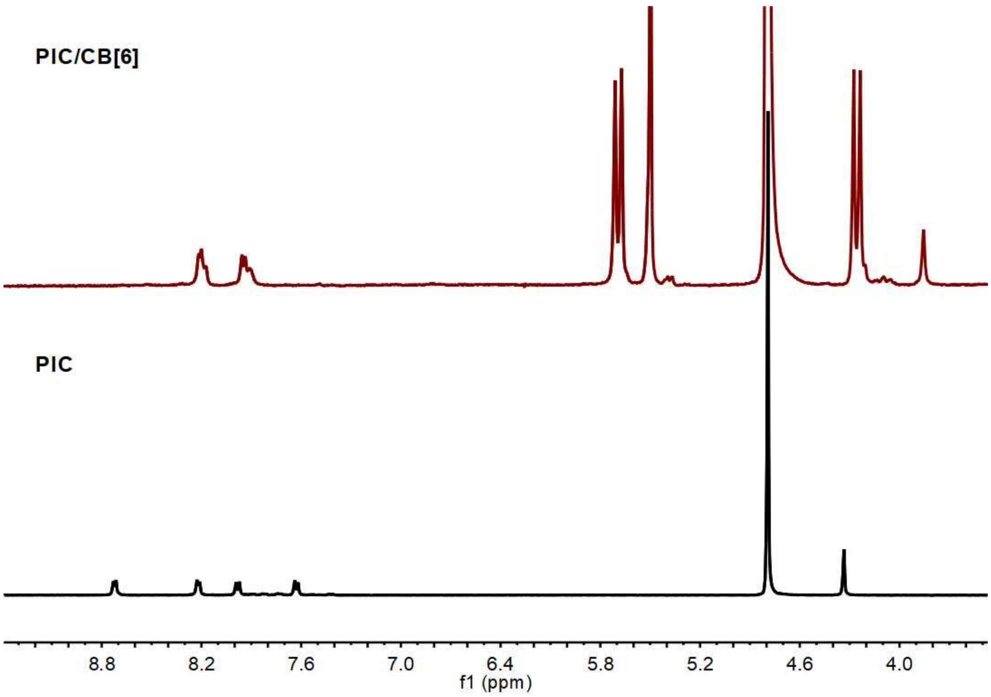

Figure S28. ^{1}H NMR (400 MHz, D_2O, 298 K) spectra of PIC/CB[6] (maroon) and PIC (black).

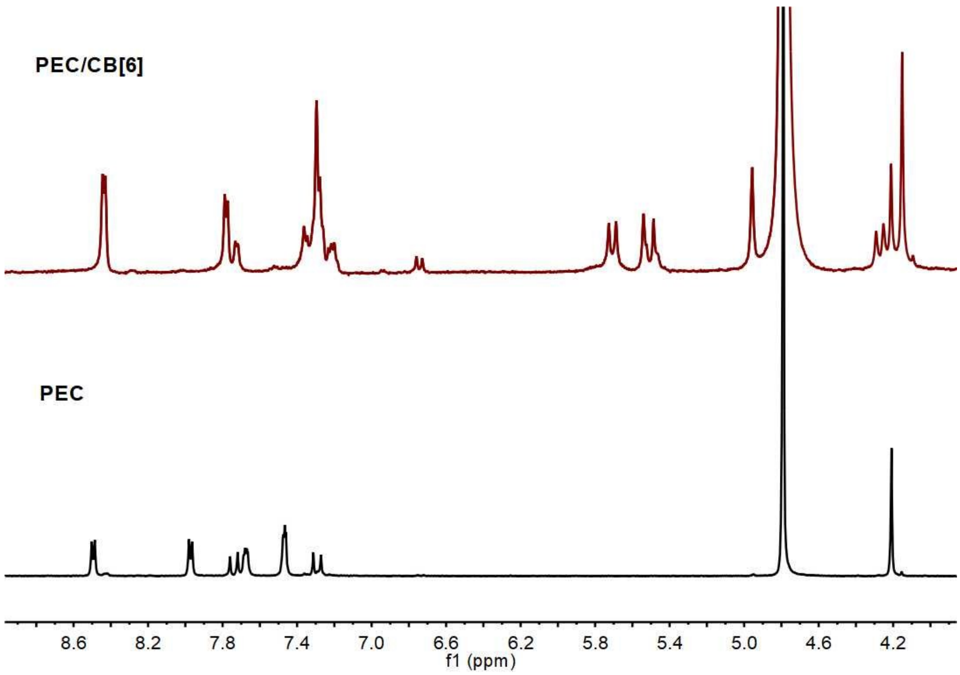

Figure S29. ^{1}H NMR (400 MHz, D_2O, 298 K) spectra of PEC/CB[6] (maroon) and PEC (black).

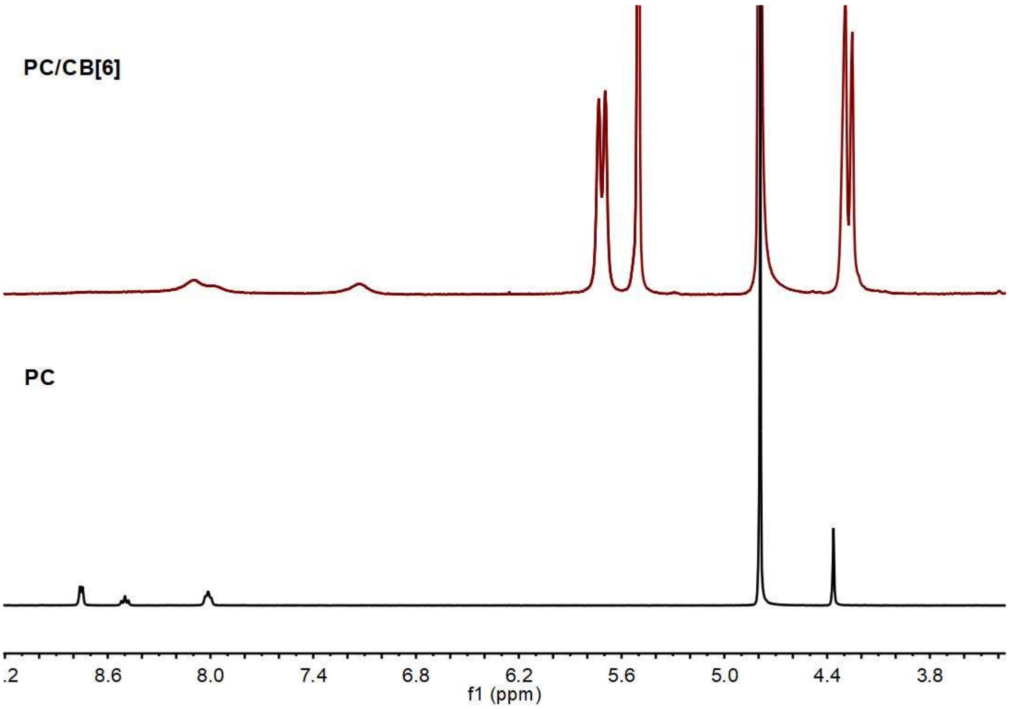

Figure S30. ^{1}H NMR (400 MHz, D_2O, 298 K) spectra of PC/CB[6] (maroon) and PC (black).

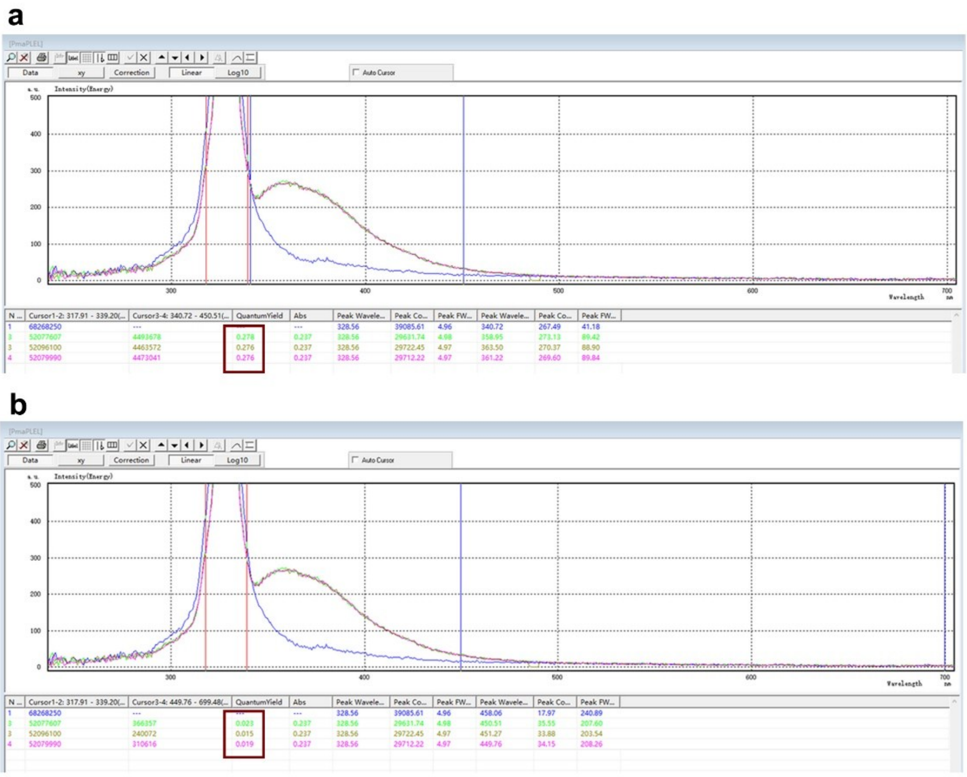

Figure S31. Fluorescence (a) and Phosphorescence (b) quantum efficiency of PFC/CB[6].

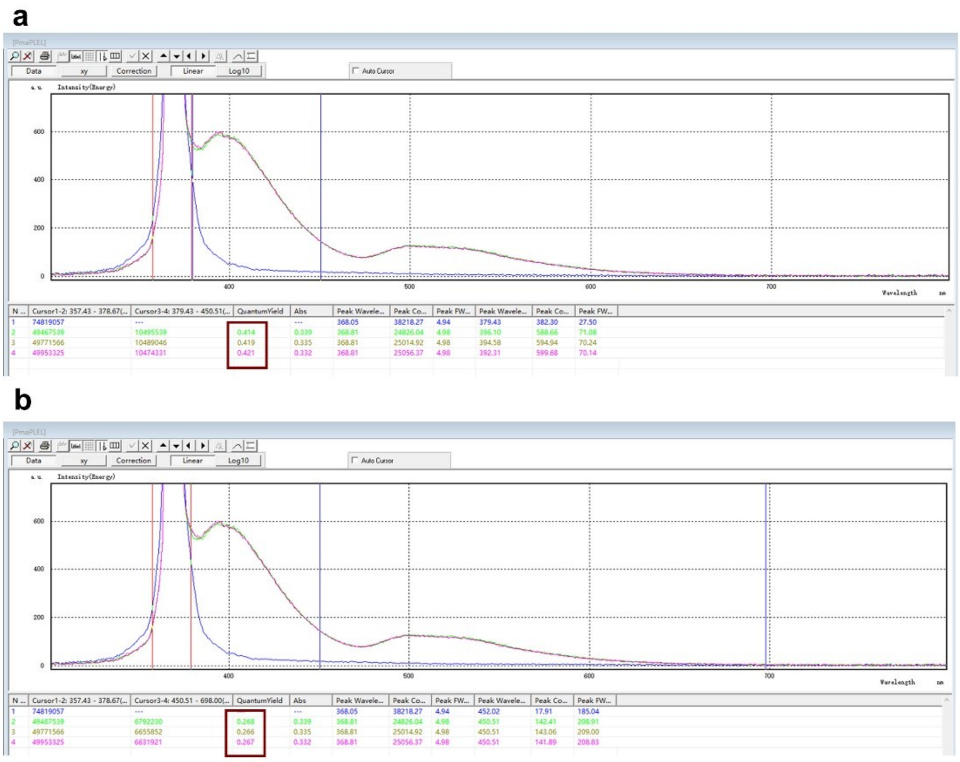

Figure S32. Fluorescence (a) and Phosphorescence (b) quantum efficiency of PCC/CB[6].

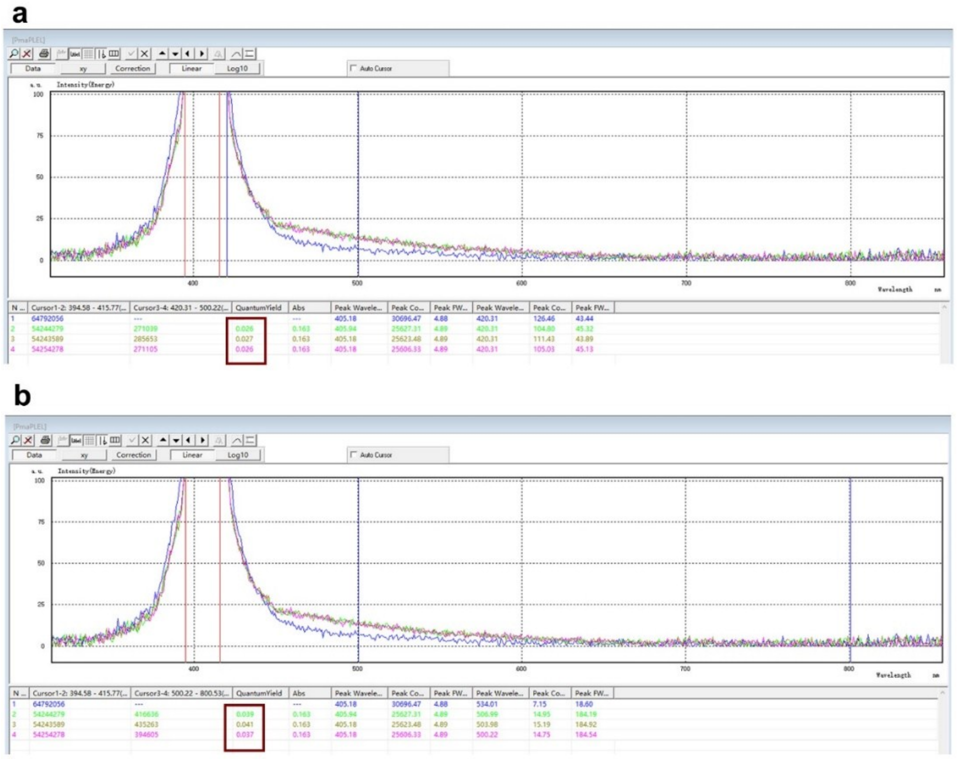

Figure S33. Fluorescence (a) and Phosphorescence (b) quantum efficiency of PIC/CB[6].
